# Unravelling Receptor and RGD Motif Dependence of Retargeted Adenoviral Vectors using Advanced Tumor Model Systems

**DOI:** 10.1038/s41598-019-54939-9

**Published:** 2019-12-06

**Authors:** M. Chernyavska, M. Schmid, P. C. Freitag, V. Palacio-Castañeda, A. Piruska, W. T. S. Huck, A. Plückthun, W. P. R. Verdurmen

**Affiliations:** 1 Department of Biochemistry, Radboud Institute for Molecular Life Sciences (RIMLS), Radboud University Medical Center, Geert Grooteplein 28, 6525 GA Nijmegen, The Netherlands; 2 Department of Biochemistry, University of Zurich, Winterthurerstrasse 190, 8057 Zurich, Switzerland; 3 Department of Physical-Organic Chemistry, Institute for Molecules and Materials, Radboud University, Heyendaalseweg 135, 6525 AJ Nijmegen, the Netherlands

**Keywords:** Biochemistry, Molecular biology

## Abstract

Recent advances in engineering adenoviruses are paving the way for new therapeutic gene delivery approaches in cancer. However, there is limited knowledge regarding the impact of adenoviral retargeting on transduction efficiency in more complex tumor architectures, and the role of the RGD loop at the penton base in retargeting is unclear. To address this gap, we used tumor models of increasing complexity to study the role of the receptor and the RGD motif. Employing tumor-fibroblast co-culture models, we demonstrate the importance of the RGD motif for efficient transduction in 2D through the epithelial cell adhesion molecule (EpCAM), but not the epidermal growth factor receptor (EGFR). Via optical clearing of co-culture spheroids, we show that the RGD motif is required for transduction via both receptors in 3D tumor architectures. We subsequently employed a custom-designed microfluidic model containing collagen-embedded tumor spheroids, mimicking the interplay between interstitial flow, extracellular matrix and adenoviral transduction. Image analysis of on-chip cleared spheroids indicated the importance of the RGD motif for on-chip adenoviral transduction. Together, our results show the interrelationship between receptor characteristics, the RGD motif, the 3D tumor architecture and retargeted adenoviral transduction efficiency. The findings are important for the rational design of next-generation therapeutic adenoviruses.

## Introduction

Currently, the field of gene therapy is witnessing a revival, with three new FDA approvals in 2017 and many novel approaches being pursued in preclinical research and clinical trials^1^. Adenoviral vectors have multiple advantages for gene therapy applications, such as high transduction efficiency in dividing and post-mitotic cells, extremely low risk of insertional mutagenesis and high payload capacity^2^. This is especially true for the so-called ‘gutless’ viruses which are free of viral genes. Human adenovirus serotype 5 (HAdV5) is the most studied adenovirus and has been extensively used as a vector for gene therapy^2,3^, but other serotypes are now being investigated as well.

HAdV5 infects cells in a two-step mechanism. The trimeric fiber knob, situated at the tip of the fibers located within the penton, interacts with the coxsackie-adenovirus receptor (CAR)^4^. Subsequently, an Arg-Gly-Asp (RGD) loop within the penton interacts with α_V_ integrins of the target cell, required for efficient virus internalization and endosomal escape^5,6^. Harnessing the benefits of HAdV5-based therapies requires overcoming several challenges: the virus must be targeted to the desired tissue, and it must be detargeted from its inherent liver tropism and protected from the immune system^2,7,8^. If a gutless virus is to be used as a basis, an efficient production system must be developed^9^.

When describing efforts that have been made to overcome these challenges, it is useful to separate them into targeting, detargeting and overcoming the immune response. To alter targeting, the knob domain has been exchanged between serotypes^10,11^, but this cannot access completely new receptors. Peptides have been displayed on the knob^12^, but this can interfere with knob assembly and will not ablate binding to the original receptor. Using an anti-knob scFv fragment fused to epidermal growth factor (EGF)^13^, retargeting to the EGF receptor (EGFR) was aimed for, but monovalent binding will be of limited affinity, and scFv fusions are notoriously aggregation-prone. In 2013, a flexible adapter system was developed based on the use of designed ankyrin repeat proteins (DARPins)^14^. Briefly, by trimerizing a knob-binding DARPin, and linking it by a flexible linker to a target-binding DARPin, a very general retargeting can be achieved, as the adapter binds very tightly, blocks binding of the knob to the original CAR, and high-affinity DARPins can be selected from very diverse libraries to bind to basically any target^15^. DARPins are small (~15 kDa) very robust proteins which fold well even as fusion proteins.

Recently, we reported *in vivo* experiments in which several approaches to target, to detarget and to avoid immune recognition were combined^7^. Targeting to different receptors was achieved by the adapter strategy^14^, liver tropism was reduced by introducing mutations in the hexon that eliminate factor X binding^16^, and by creating a high-affinity protein coat based on a trimerized scFv fragment binding to the hexon, the immune recognition was decreased in addition to a further decrease in liver tropism^7^. In the absence of the EGFR- or Her2-targeting adapter, it was observed that murine fibroblasts and fibrocytes were transduced when the virus was injected intratumorally.

While binding to integrins is not required for the cell association of the virion^17^, the RGD motif may contribute to fibroblast transduction by adenoviral vectors. Integrin expression is ubiquitous and particularly high on fibroblasts^18^. It is thus important to establish whether the association of the RGD motif with integrins is necessary for the infection process also when receptors other than CAR are being used for initial cell association. In this study, we therefore investigated the role of the RGD motif in transduction with adenoviral vectors retargeted to human EGFR and epithelial cell adhesion molecule (EpCAM), both on tumor cells and on fibroblasts, using the adapter strategy described above^7,14^. We utilized both 2D monolayer tumor-fibroblast co-cultures and 3D spheroid co-culture models. The use of 3D models is particularly relevant in this context, as integrin expression levels, accessibility and downstream signaling activity can differ widely between conventional 2D and more complex 3D systems that can contain extracellular matrix materials^19–22^. On the other hand, for mechanistic investigations regarding the role of the RGD motif, 3D model systems are much more tractable than *in vivo* models. We also employed a tumor-on-a-chip microfluidic device designed for capturing spheroids. Microfluidic devices can be employed to experimentally model tumor-specific features in a controlled manner, including the presence of multiple cell types, extracellular matrices (ECM), concentration gradients, fluid flow as well as the addition of therapeutic agents to the tumor^23^. Using co-culture models in combination with optical clearing of 3D tumor cell cultures, we demonstrate the receptor-dependent role of the RGD motif and its different role in 2D and 3D cultures.

## Results

### Luciferase assay: cell line screening and model selection

To select appropriate models for the investigation of the role of the RGD motif at the penton base in transduction with retargeted adenoviral vectors in 3D static and microfluidic tumor models, we first screened individual cell lines by retargeting HAdV5 towards different tumor cell markers. These cell lines would then be used in co-culture models. We compared the CAR-mediated transduction of naked virus (both as a WT with intact RGD and ΔRGD) to transduction with retargeted viruses by DARPin-based retargeting adapters^14^ to either Her2, EGFR, Her3 or EpCAM, again using WT and ΔRGD viruses.

For this purpose, we used a series of tumor cell lines that have been described in the literature to express one or several of these receptors (Supplementary Table S1), as well as two primary fibroblast cell lines: IMR-90 (lung) and C5120 (skin).

We aimed to identify two co-culture models for subsequent studies: one model where both fibroblasts and tumor cells would be transduced and a second model where preferentially tumor cells would be transduced. A model where both tumor cells and fibroblasts are transduced would allow us to investigate factors arising from either the 3D tumor context or the virus structure that are important for transduction of both cell types. In contrast, the second model could serve as a lead for generating next-generation adenoviral delivery systems with a further enhanced specificity regarding the transduction of tumor cells. Using luciferase-encoding virus, we measured luciferase activity 48 h after transduction of 2D monolayer cultures of a series of tumor cell lines.

In tumor cells, use of a DARPin adapter targeting to an overexpressed receptor led mostly to an increased transduction, compared to the non-binding DARPin E2_5 (Supplementary Fig. S1).

Interestingly, rather different consequences regarding the effect of the deletion of the RGD motif in the penton base were observed depending on which receptor was targeted. For CAR-mediated transduction (in the absence of any adapter), a pronounced decrease in transduction level by ΔRGD was observed only in C5120 cells, while virtually no decrease was observed in the remaining tested cell lines. If the virus is coated with an adapter that blocks all binding interactions via the knob protein and carries the non-targeting control E2_5 DARPin, strongly decreased transduction efficiency of ΔRGD viruses compared to the WT virus was observed for all cell lines except for SKOV-3 cells, where only a minor decrease in transduction efficiency was observed. Remarkably, in MCF-7 cells (Supplementary Fig. S1), the knob-blocked ΔRGD virus gave no detectable signal, using the highly sensitive luciferase assay, while transduction was detected for the knob-blocked WT virus and for the non-blocked ΔRGD virus. The transduction with the knob-blocked WT virus is in line with previous observations of essentially no CAR expression and an important role of αvβ5 integrins in AdV transduction of MCF-7 cells^24^. The interaction partner on the virus that rescues transduction with non-blocked ΔRGD virus (which is expected to transduce only through CAR and not through αvβ5 integrins) remains undefined, but could be either residual CAR or yet another unidentified interaction partner of the knob.

When comparing transduction through the tumor marker-specific adapters, we observed that transduction through EpCAM was lower for the ΔRGD virus in all tumor cell lines as well as in both fibroblast cell lines (P < 0.0001 for the effect of the ΔRGD modification on EpCAM-retargeted virus across all tested cell lines, vs. P = 0.0002 for the decreased activity of the ΔRGD modification on non-retargeted virus). For retargeting to the other receptors, no strong trends were observed. Of note, EGFR-mediated transduction was very high in A-431 cells and in both fibroblast cell lines, and showed no decreased transduction efficiency for the ΔRGD viruses. This suggests that the high efficiency seen for EGFR-mediated adenoviral transduction is not dependent on the interaction of the RGD motif with integrins.

Based on these data, we decided to continue with two co-culture models: first, A-431 and C5120 cells, which can both be efficiently transduced with EGFR-retargeted HAdV5, where there is no apparent effect of the RGD motif; second, MCF-7 and C5120, where tumor cells are preferentially transduced and where the RGD motif contributes considerably to the transduction efficiency. We quantified receptor levels of EpCAM and EGFR on these cell lines, and could confirm that for EGFR, levels were very high on A-431 cells, moderate-to-low on C5120 fibroblasts and low on MCF-7 cells (Supplementary Fig. S2). For EpCAM, levels were highest on MCF-7 cells, followed by A-431 cells; levels were very low on C5120.

### Transduction efficiency of retargeted HAdV5 in co-cultures in 2D

We used wild-type and ΔRGD viruses encoding iRFP670 as a reporter in co-cultures of individually labelled cell types, in order to be able to identify the transduced cell type by means of flow cytometry and confocal microscopy. Employing monocultures as a reference, we wished to evaluate potential effects on transduction of mutual competition between the cell lines, for example for binding sites, due to the markedly higher receptor expression levels on tumor cells than on fibroblasts (Supplementary Fig. S2).

**Figure 1:**
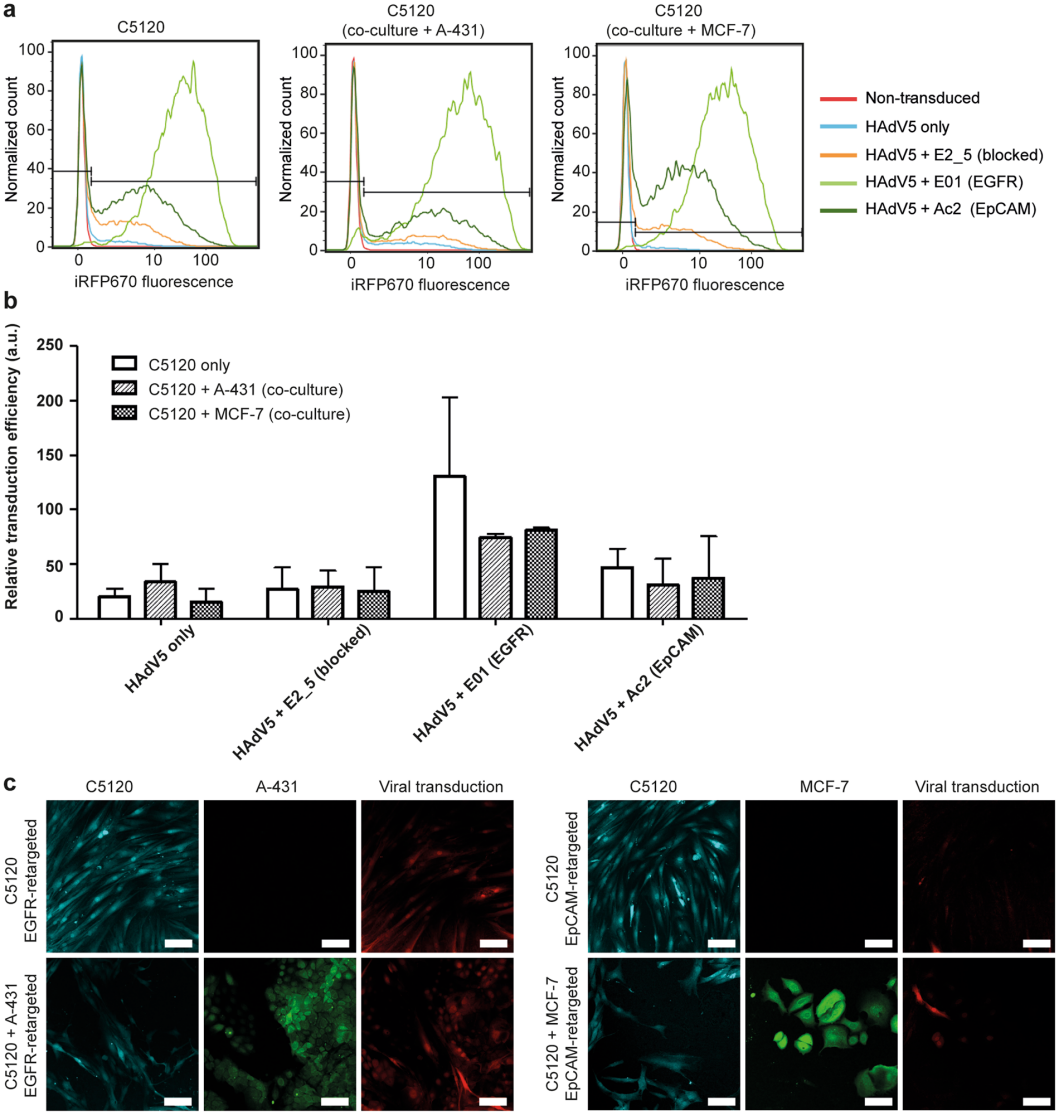
Flow cytometry and confocal microscopy of HAdV5-transduced fibroblast mono- and co-cultures. (**a**) Overlay histogram plots of the normalized cell count of the iRFP670-encoding WT HAdV5-transduced fibroblasts are shown. HAdV5 was retargeted to EGFR and EpCAM with E01 and Ac2 DARPin adapters, respectively. HAdV5 only (i.e. CAR-directed) and HAdV5 complexed with the non-binding DARPin E2_5 (i.e. knob-blocked) were used as controls. Representative plots are shown, n = 3. (**b**) Flow cytometry-based quantification of transduced fibroblasts in monoculture or in co-culture with tumor cells as in (**a**). A two-way ANOVA showed no significant effect of the culture condition (i.e. mono- vs. co-culture; P = 0.1926). Bars show relative transduction ± s.e.m., n = 3. (**c**) Confocal microscopy images of infection by EGFR-retargeted and EpCAM-retargeted HAdV5 are shown. C5120 fibroblasts were labelled with CellTrace Yellow (cyan), A-431 and MCF-7 cells were labelled with CFSE (green). iRFP670 was used for detection of virus-transduced cells. Representative images from two independent experiments with three images taken per condition per experiment are shown. The scale bars are 100 µm.

Corroborating findings from the luciferase assay, we observed in fibroblasts the highest transduction efficiency with the EGFR-retargeted virus using flow cytometry (see Fig. 1a,b for WT, Supplementary Table S2 for ΔRGD), followed by transduction via retargeting to EpCAM and transduction with the virus only (presumably infecting via CAR). Surprisingly, a marginally higher transduction was observed for E2_5-blocked virus as compared to virus only, which was evident for both the WT and ΔRGD virus. In contrast, the luciferase assay only showed an increase for the ΔRGD virus (Supplementary Fig. S1). A possible explanation for these findings is that the non-targeting control DARPin E2_5 non-specifically interacts with C5120 cells, leading to a relatively efficient transduction.

No statistically significant differences in trends were observed between fibroblast mono- and co-cultures, suggesting there was no meaningful competition of the virus for receptors given the high levels of receptors present on the tumor cells. The results of the flow cytometry analysis were qualitatively supported by confocal microscopy images of the mono-culture and co-culture models transduced with EGFR- and EpCAM-retargeted adenovirus (Fig. 1c).

**Figure 2:**
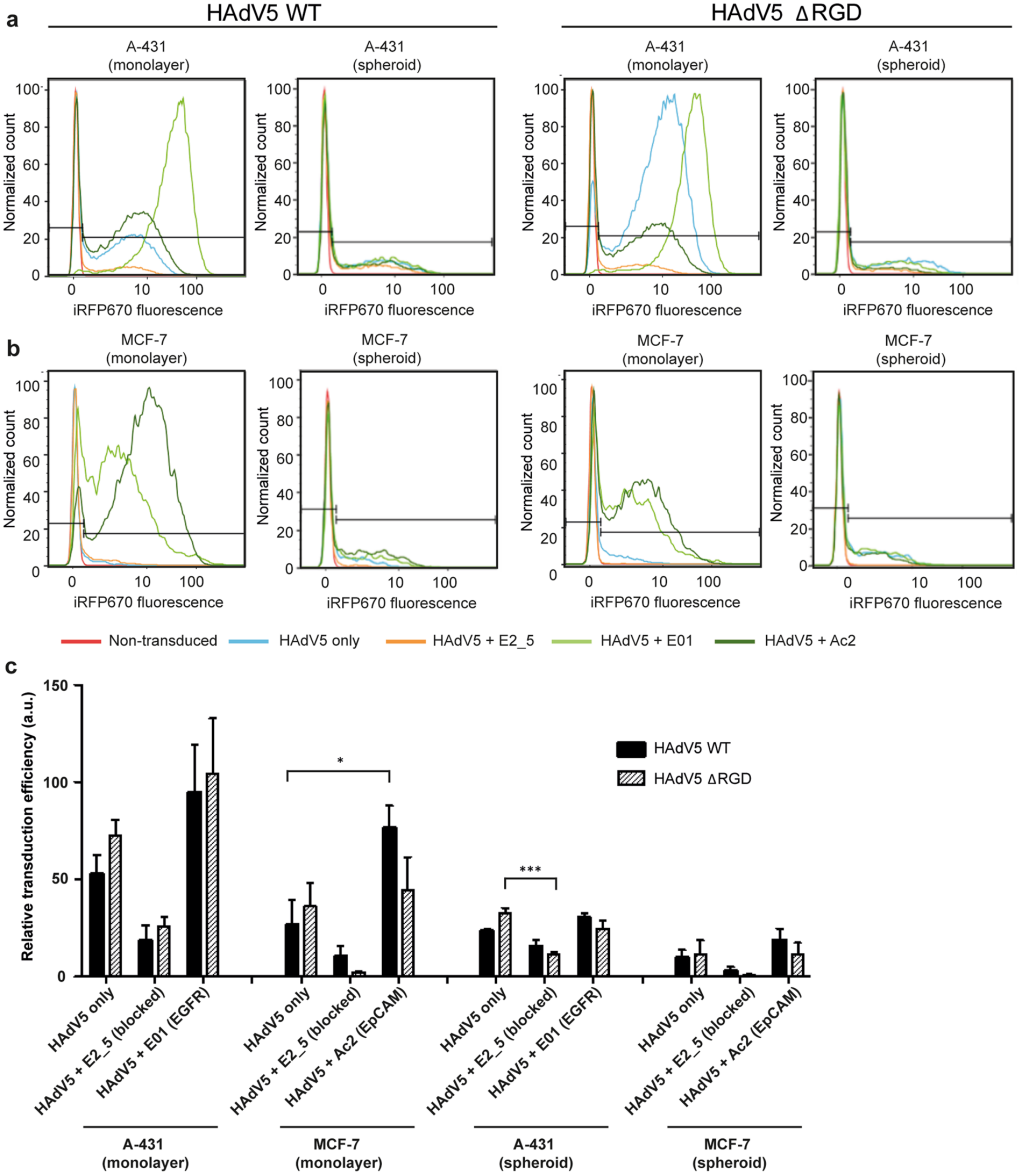
Flow cytometry of transduced tumor cells from co-cultures with fibroblasts in monolayer and spheroids. Cells were transduced with WT HAdV5 and ΔRGD HAdV5. (**a**) Overlay histogram plots of the normalized cell count of iRFP670-encoding WT and ΔRGD HAdV5-transduced A-431 cells are shown. HAdV5 was retargeted to EGFR with the DARPin E01 adapter, and to EpCAM with the DARPin Ac2 adapter. HAdV5 only (i.e. CAR-directed) and HAdV5 complexed with the non-binding DARPin E2_5 adapter (i.e. knob-blocked) were used as controls. Representative plots are shown, n = 3. (**b**) Same as described in (**a**) for MCF-7 cells co-cultured with C5120 fibroblasts. (**c**) Flow cytometry-based quantification of tumor cells transduced with WT or ΔRGD viruses. Statistical analysis by two-way ANOVA showed transduction in 3D cultures was significantly lower than transduction in 2D cultures for A-431 (WT: P = 0.0045; ΔRGD: P = 0.0008) and MCF-7 cells (WT: P = 0.014; ΔRGD: P = 0.0246). Bars show relative transduction ± s.e.m., n = 3. *indicates P < 0.05; ***indicates P < 0.001.

Similarly to the luciferase assay results, using flow cytometry we noted essentially no reduction for EGFR-mediated transduction efficiency with the ΔRGD mutant in comparison to the virus with the RGD motif in tumor cells from 2D fibroblast-tumor co-cultures (Fig. 2a,c). In contrast, a considerable decrease upon deletion of the RGD motif was again observed for the EpCAM-retargeted transduction efficiency in MCF-7 cells (Fig. 2b,c).

### Transduction efficiency of retargeted HAdV5 in co-cultures in 3D

Next, we investigated the viral transduction efficiency and specificity in 3D spheroid cultures grown in static conditions (in the absence of flow). Static spheroid co-cultures mimic non-vascularized tumor regions with little or no fluid flow present, thus making nutrient and therapeutic delivery dependent exclusively on diffusion^25^. The viral retargeting effect was also observed in 3D for WT viruses, although generally transduction efficiencies were far lower in 3D than in monolayer cultures (Fig. 2 shows results for tumor cells, Supplementary Table S3 for fibroblasts). In monolayer cultures, EGFR retargeting led to the highest mean HAdV5 transduction efficiency for A-431 cells, followed by fibroblasts. In 3D, however, the EGFR-retargeted transduction efficiency was greatly decreased for both A-431 cells and fibroblasts, emphasizing the importance of 3D models as well as of geometric factors of access to the cells. In A-431 cells, transduction via CAR or EpCAM showed similar trends of more efficient transduction in monolayers (Fig. 2a,c). In 3D co-cultures with MCF-7 and C5120 cells, comparable trends were observed for the tumor markers (Fig. 2b,c, Supplementary Table S3), but CAR-mediated transduction was low in all conditions.

Significantly lower transduction efficiencies were also observed for ΔRGD viruses in 3D cultures compared to 2D cultures for all conditions, as quantified by flow cytometry (Fig. 2). In contrast to observations made in 2D cultures, however, transduction by ΔRGD viruses was generally lower in spheroids for retargeted, non-retargeted and blocked viruses, though different trends were observed for the different cell lines and targeted receptors (Fig. 2, Supplementary Table S3). In tumor cells, deletion of the RGD motif resulted in a mild reduction in transduction efficiency with EpCAM-retargeted virus in A-431 cells and a moderate reduction in MCF-7 cells (Fig. 2). A less pronounced reduction was seen for the EGFR-retargeted virus, with the relative transduction efficiency being similar in both A-431 and MCF-7 cells.

Major reductions by the RGD motif deletion were, however, observed in fibroblast transductions (Supplementary Table S3). The transduction efficiency decreased greatly in co-cultures with A-431 cells with EGFR- and EpCAM-retargeted virus. In MCF-7 co-culture spheroids, the EpCAM-mediated transduction efficiency of fibroblasts with ΔRGD virus was much lower as compared to the WT virus. Clearly, the reduction in the transduction efficiency of EpCAM-targeted ΔRGD viruses was more pronounced in 3D cultures as compared to 2D cultures, suggesting independently contributing effects of both the type of receptor and the tumor architecture. These findings underline the importance of 3D models for investigating transduction dependency in context with viral modifications.

Remarkably, in this 3D set-up, the transduction efficiency of targeting to the original receptor (i.e. CAR) resulted in comparable numbers of transduced tumor cells as with EGFR- and EpCAM-retargeted viruses. This contrasts to what was seen in 2D cultures, where CAR-mediated transduction was much less efficient.

### Visualization of transduction in optically cleared spheroid co-cultures

**Figure 3:**
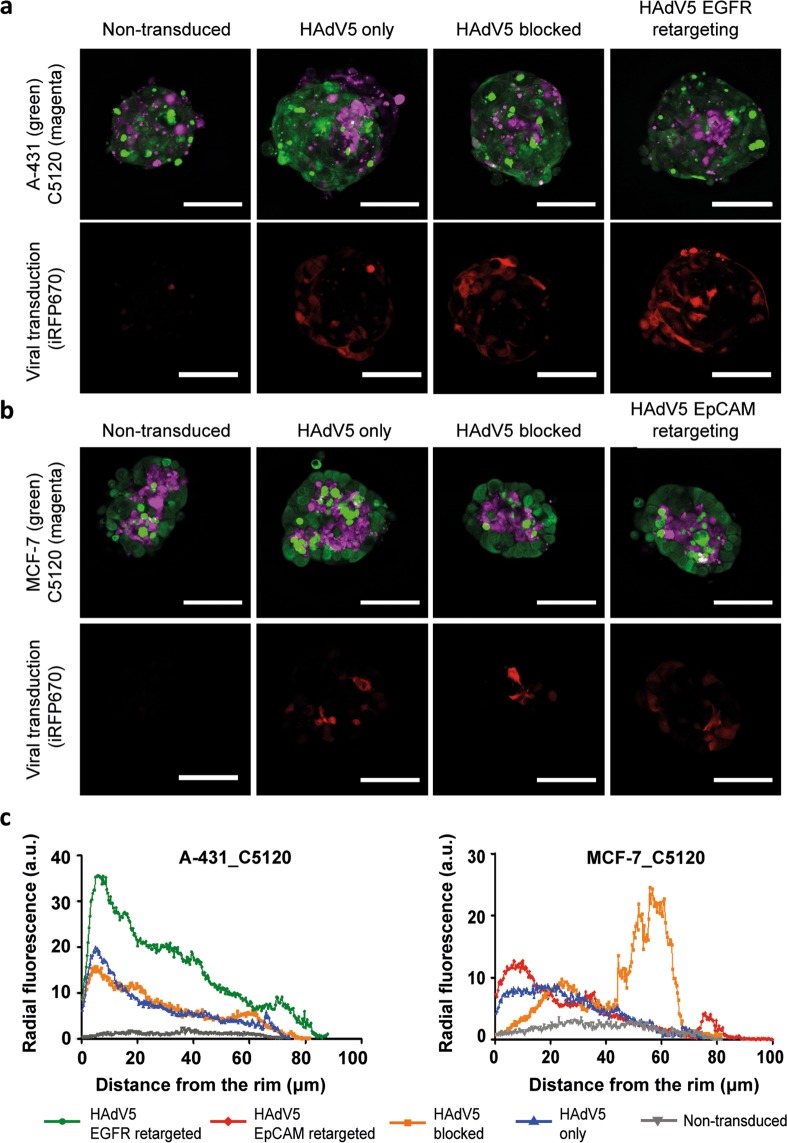
Transduction of tumor spheroids with HAdV5 directed to EGFR or EpCAM. Confocal microscopy images of optically cleared A-431_C5120 (**a**) and MCF-7_C5120 (**b**) tumor spheroids (confocal slice from the centre) are shown, with the corresponding radial intensity profiles (**c**). C5120 fibroblasts were labelled with CellTrace Yellow (magenta), A-431 and MCF-7 cells were labelled with CFSE (green). iRFP670 was used for detection of the virus-transduced cells (red). EGFR retargeting was accomplished using the E01 adapter, EpCAM retargeting via the Ac2 adapter and the HAdV5 was blocked using the non-binding E2_5 adapter. Representative images are shown from two independent experiments, with three spheroids analysed per condition per experiment. The scale bars are 100 µm. (**c**) The fluorescence intensity of iRFP670 in the spheroids is represented via a radial intensity profile. The average fluorescence was plotted from the outer edge of the spheroid (0 μm) to the spheroid core. At least two spheroids measured per condition per experiment were analyzed. Two independent experiments were performed. EGFR, epidermal growth factor receptor. EpCAM, epithelial cell adhesion molecule.

To observe the transduction of cells in their native architecture in co-culture 3D tumor spheroids, confocal imaging of spheroids was performed using a SeeDB-derived optical clearing protocol that is non-toxic and compatible with most fluorescent dyes, including fluorescent proteins^26^. In Fig. 3 and Supplementary Fig. S3, confocal microscopy of tumor cells and fibroblasts transduced with WT and ΔRGD HAdV5 is presented. In Supplementary Fig. S4, sequential optical confocal slices of transduced co-culture tumor spheroids are shown that illustrate that fluorescence could be observed throughout the entire spheroid after the clearing protocol.

Generally, the confocal images corroborate the findings from flow cytometry (Figs.  2, 3ab). Interestingly, tumor cells and fibroblasts cluster together, rather than forming a homogenous distribution in the spheroids. The clusters of fibroblasts were localized more towards the centre of the spheroids in both A-431_C5120 and MCF-7_C5120 co-culture models, similar to what has been observed before in other spheroid co-cultures^27–29^. The localization of fibroblasts preferentially in the centre may have contributed to the very low transduction levels of fibroblasts that were observed in the flow cytometry analyses of co-culture tumor spheroids (Supplementary Table S3).

Unsurprisingly, the images indicate that the transduction of tumor spheroids is predominantly at the rim, although transduction of more centrally localized cells was observed as well, as can be seen from a quantitative analysis of rim-to-core fluorescence intensities (Fig. 3c) as well as Z-stacks of confocal images of the different conditions (Supplementary Fig. S4). In A-431_C5120 spheroids, we observed a gradual decrease of intensity towards the core. Similar observations were made for transduction with ΔRGD viruses (Supplementary Fig. S3). In MCF-7_C5120 spheroids, a comparable trend was observed, though a second peak for the knob-blocked viral transduction signal was seen more centrally, which is mainly due to transduction of the fibroblasts located in the core of the spheroid (Supplementary Fig. S5). This indicates the ability of the viral particles to diffuse to the spheroid core in the absence of a binding site barrier caused by a high density of receptors on the cells in the outer rim of the spheroid.

Confocal microscopy images of the monolayer co-cultures and spheroid co-cultures provide further insights regarding the differences arising from deleting the RGD motif (Supplementary Fig. S3). In 2D co-cultures, decreases in transduction efficiency for ΔRGD compared to WT were seen for EpCAM-targeted viruses in MCF-7_C5120 co-cultures. Contrasting this, in 3D co-cultures, decreases for both EpCAM- and EGFR-targeted ΔRGD variants were observed, though decreases were still much more pronounced for EpCAM-directed transduction in MCF-7_C5120, compared to EGFR-directed transduction in A431_C5120 (Supplementary Fig. S3).

### Effect of flow and extracellular matrix on viral transduction in tumor-on-a-chip

**Figure 4:**
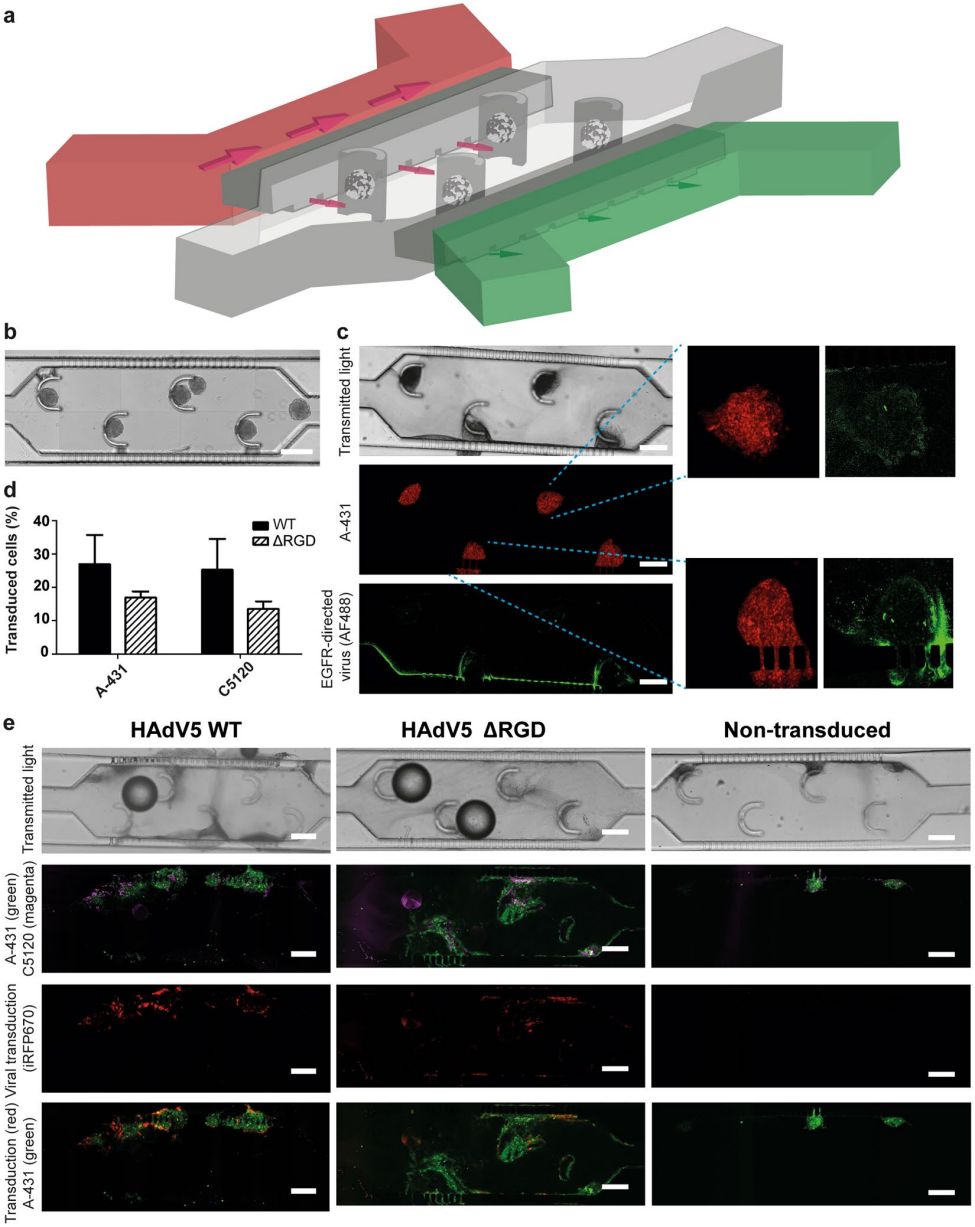
A tumor-on-a-chip device designed for capturing tumor spheroids and investigating viral transduction on-chip. (**a**) Schematic 3D representation of the tumor-on-a-chip device. The main tumor compartment with spheroid-capturing posts is indicated in grey, with capillary and lymph-mimicking channels in red and green, respectively. The outlet of the capillary-mimicking channel is connected to a tunable resistance chip (See Supplementary Fig. S6). Not drawn to scale. Supplementary Fig. S6 provides a drawing to scale. (**b**) Reconstructed image of the tumor compartment of the tumor-on-a-chip device freshly loaded with co-culture spheroids. The scale bar is 300 µm. (**c**) On-chip visualization of the trafficking of the retargeted adenovirus. Confocal microscopy images of tumor spheroids embedded in collagen on-chip perfused with Alexa Fluor 488-labelled, EGFR-retargeted adenovirus are shown. Tumor cells were stained with CellTrace Far Red. The green ridge indicates the interface between medium and the collagen gel where viral particles accumulate. Insets indicate viral particles enriched on the outer rim of the spheroids. The scale bar is 300 µm. High-resolution images of the viral particle enrichment at the spheroid rim are provided in Supplementary Fig. S7. (**d**) Quantification of the cellular transduction on-chip. The fraction of the virus-transduced A-431 and C5120 cells was determined via image analysis using Fiji software and plotted. Error bars reflect the s.e.m, n = 3. (**e**) On-chip transduction of tumor spheroids with EGFR-retargeted HAdV5 with or without the RGD motif. Confocal microscopy images of on-chip optically cleared tumor spheroids transduced with EGFR-retargeted HAdV5 (with and without RGD) and non-transduced controls are shown. C5120 fibroblasts were labelled with CellTrace Yellow (magenta), A-431 cells were labelled with CFSE (green). iRFP670 (red) was used for detection of the virus-transduced cells. Representative images from three independent experiments are shown. The scale bars are 200 µm.

Subsequently, we investigated transduction with viral vectors in a microfluidic tumor-on-a-chip model. In contrast to the non-vascularized tumor model described above (tumor spheroids), the tumor-on-a-chip system was developed to model a perfused tumor. Small cross-channel connections in our design allow basic resemblance to a leaky vasculature in a model where the hydrostatic pressure can be controlled (Fig. 4a). We designed the device to capture spheroids on-chip in a collagen matrix, with separate channels for an inlet (i.e. capillary-mimicking) and outlet (i.e. lymph-mimicking) flow to mimic blood supply and lymph drainage. Devices were loaded with a suspension of spheroids in collagen, and spheroids were captured by the corresponding elements in the main device compartment (Fig. 4b). A separate microfluidic device with tunable resistance was used to induce and adjust the interstitial flow through the tumor compartment (Supplementary Fig. S6).

To evaluate the trafficking of viral particles through the collagen matrix, we perfused EGFR-retargeted, Alexa Fluor 488-labelled viral particles through the chip containing collagen with A-431_C5120 spheroids (Fig. 4c). Using confocal image analysis, we quantified the intensity profile in multiple regions of interest (ROI) of the tumor compartment to analyse the viral particle distribution in the microfluidic device and its enrichment around spheroids (Supplementary Fig. S7). We observed a strong accumulation of the viral particles at the entry site into the collagen matrix, which could be explained by the formation of a ‘filter cake’, where a high concentration of viral particles at the collagen interface limits particle infiltration into the collagen. Beyond this initial layer, viral particles were distributed relatively evenly throughout the collagen, with a clear enrichment at the border of the spheroids (Fig. 4c, Supplementary Fig. S7), likely due to the interaction of the EGFR-targeting DARPin E01 adapter with EGFR expressed on cells in the spheroid.

Finally, we compared the viral retargeting to EGFR on-chip in an A-431_C5120 co-culture model of HAdV5 with and without the RGD motif (Fig. 4d,e). Following a 48-h perfusion, spheroids spread through the compartment and transduction was observed throughout the compartment, even though it was absent in the remaining core regions of the tumor spheroids, similar to what was seen under static conditions (Fig. 4e). A high-magnification image of the transduction with the WT and ΔRGD EGFR-retargeted virus on-chip shows limited transduction in spheroid cores (Supplementary Fig. S8). In contrast to the fibroblast localization in the tumor spheroids in the absence of flow, after 48 h in the microfluidic chip in the presence of flow, the collagen-embedded spheroids were significantly spread out and there was no evident enrichment of fibroblast in the remaining centre. We observed a non-significant trend towards lower efficiency of transduction with the ΔRGD virus than with the virus with the WT RGD, which is in line with the small differences that were observed when comparing WT versus ΔRGD viruses in static spheroids with EGFR-retargeted viruses (Fig. 4d, cf. Fig. 2). The method to quantify transduction on-chip on the basis of confocal images was validated through analysing results of an identical experiment by both microscopy and flow cytometry, yielding comparable results (Supplementary Fig. S9).

## Discussion

At present there is still an insufficient understanding of the principles that underlie the transduction of adenoviruses retargeted to novel receptors, and how complex (tumor) architectures are involved. Furthermore, it is unclear what the interacting partners are for adenoviral transduction in the absence of any cognate receptor. In this study, using 2D and several 3D tumor models, we focused on the role of the integrin-binding RGD motif. Specifically, we investigated the role of the RGD motif in transduction when receptors other than CAR are being used for cell association, or when no receptor is targeted (and CAR targeting is blocked), in various types of fibroblast-tumor cell co-cultures. We demonstrate that removing the RGD motif in the capsid of retargeted adenoviral vectors has different consequences depending on which receptor is targeted. We also show that WT and ΔRGD viruses behave differently in 3D and 2D cultures, with ΔRGD viruses consistently showing a lower relative transduction in 3D when compared to WT viruses. In 2D cultures, this was receptor- and cell-line dependent.

We selected two tumor co-culture cell models, using EGFR and EpCAM as targetable receptors on tumors. EpCAM is abundantly expressed in primary tumors and metastases of most epithelial malignancies, but also in normal epithelia, albeit at lower levels and mainly in a basolateral location with sequestration in intercellular boundaries, where it is less accessible^30^. EGFR is overexpressed on many tumors, but at lower levels also on normal tissues and fibroblasts^31,32^. The tumor cell models we chose have been reported to be either low (MCF-7)^24^ or low to moderate (A-431)^33^ with respect to the natural receptor CAR, and exhibit therefore relatively low infection efficiency by WT HAdV5.

In monolayers, EGFR-directed adenoviral transduction was independent of the RGD motif (Fig. 2, Supplementary Figs. S1 and S3). This observation regarding EGFR in monolayer cultures is in line with a previous observation using adenoviruses retargeted to EGFR using a fusion protein containing epidermal growth factor, where the authors showed that a GRGDSP peptide could block transduction with WT adenoviruses, but not of EGFR-retargeted adenoviruses^13^. In contrast to the EGFR finding, for EpCAM retargeting a dependency on the RGD motif was observed in 2D tumor cell models (Fig. 2, Supplementary Figs. S1 and S3).

For transduction with WT adenoviruses via CAR, it is well known that the penton base and fiber play central roles in the initial uncoating and disassembly of adenoviruses. A drifting motion of CAR bound to the fiber in combination with binding of the penton base to immobile integrins via the RGD motif is believed to facilitate the extraction of the fiber from the viral capsid, thereby exposing the endosomolytic protein pVI^6^, which induces positive membrane curvature leading to endosomal escape^34^.

Shayakhmetov *et al*. investigated the effect of deletion of the RGD motif on the adenoviral transduction of HAdV5, which uses CAR as a primary attachment receptor, and HAdV35, which uses CD46 as a primary attachment receptor^35^. They observed that regardless of the primary attachment receptor, the RGD deletion did not influence virus attachment, though significantly reduced viral internalization rate^35^, which would be in agreement with the model described above. Previous investigations of the mechanism of adenovirus infection have thus focused mainly on initial binding of adenoviruses to natural receptors, i.e. CAR and CD46, each of which can be exploited by a different subset of adenoviruses serotypes^35,36^. Here we mediated entry via receptors of rather different types, EpCAM and EGFR, whose behaviour with respect to internalization differs significantly. Whereas the internalization rate of EGFR is particularly high (0.08 min^−1^)^37^, the reported internalized fraction of 18.7% after 90 min for EpCAM appears to reflect a much slower internalization rate^38^.

Entry via EpCAM appears to require a similar mechanism as entry via CAR, suggesting a required conformational change of the penton base to expose the protein VI for viral internalization and endosomal escape and participation of the RGD motif and integrins. In contrast, in case of the EGFR-mediated transduction, association of the knob-adapter complex with EGFR could already be sufficient to induce receptor internalization and pVI exposure with concomitant endosomal escape, thus not requiring the RGD motif. Whether this process induces a similar change in the penton base or whether subsequent endosomal escape employs different mechanisms will have to be the subject of future studies.

In 3D spheroids, a lower efficacy of ΔRGD viruses compared to WT viruses was not only seen for EpCAM retargeting, but also for EGFR retargeting. As mentioned above, for EGFR, this was not the case in 2D. Especially for fibroblasts, relative transduction via all receptors was greatly reduced for ΔRGD viruses compared to WT viruses (Supplementary Table S3). Since fibroblasts reside mostly in the core in 3D spheroid cultures, the most likely explanation is that the ΔRGD viruses have a reduced ability to access the receptors in the first place. Since fibroblasts express lower amounts of all the receptors under study than the tumor cells, the uptake by fibroblasts would be expected less efficient, and in the context of a smaller number of virions arriving there, a participation of integrins is conceivable.

Alternatively, it cannot be excluded that RGD-containing viruses interact with a different subset of integrins in 2D versus 3D, possibly leading to a relatively higher transduction efficiency in 3D for WT viruses compared to ΔRGD viruses. A mildly lower efficiency of the EGFR-retargeted ΔRGD virus compared to the EGFR-retargeted WT virus was also observed in the microfluidic tumor model (Fig. 4d).

We chose to focus on 3D systems for investigating the effect of removing the RGD motif because of known differences in the subset of integrins that are expressed in 2D and 3D culture systems as well as their functional differences. Integrins are obligate dimers consisting of an α and a β subunit that connect the extracellular matrix to the intracellular cytoskeleton. All five α_V_ integrins, two β_1_ integrins (α_5_, α_8_) and α_IIb_β_3_ recognize ligands containing an RGD motif, which are present on a large variety of integrin ligands^39^. In 2D cell cultures on plastic substrates, cells massively upregulate the levels of β_1_ and several α integrin subunits in an attempt to establish sufficient cell-matrix interactions to maintain survival^22^. In 3D, but not in 2D, a transdominant inhibition of α_v_β_3_ by a_5_β_1_ is caused by the high levels of integrin binding to fibronectin in a fibrillar matrix^19^. Hence, binding to fibronectin (which contains an RGD motif) depends on different integrins in 2D (α_v_β_3_ and a_5_β_1_) versus 3D (α_5_β_1_ only). The different integrin signature is associated with enhanced biological activities of integrin-ligand interactions in 3D^40^. For example, in 2D there is lack of the ability of integrins to bidirectionally cross-modulate between β_1_ integrins and EGFR signaling via the mitogen-activated protein kinase pathway, as observed in a 3D basement membrane assay^20^.

Moreover, CAR expression in 3D is dependent on normal tissue physiology. A cancerous microenvironment has been shown to disrupt physiological interactions with the extracellular environment, leading to an altered subcellular distribution and higher overall CAR levels, increasing the susceptibility towards adenoviral infection^41^. For EpCAM, though tumor cells express EpCAM more homogenously and more accessibly as compared to normal epithelia^30^, tumor growth in 3D is associated with reduced EpCAM levels, which was hypothesized to be related to an epithelial-mesenchymal transition (EMT)^42^. Similarly, a consistently decreased EGFR expression was seen across a range of tumor cell lines when grown in 3D versus 2D^43^. Thus, the distinct receptor expression profiles in 2D versus 3D as well as the functional consequences thereof underscore the need to investigate potential effects of removing the RGD motif in adenoviral vectors in 3D cell culture systems, illustrated by the differential uptake patterns of EGFR-retargeted ΔRGD viruses versus WT viruses in 2D and 3D systems.

Furthermore, accessibility of target cells is an important aspect that is fundamentally different in 2D, where there is no barrier, compared to 3D, where the matrix and cells represent a physical barrier^25,44^ and the presence of a high density of ligands may represent an additional binding-site barrier (BSB)^45^. The BSB occurs when tight interactions between targeted viral particles and cells (e.g. via avidity effects) prevent deeper penetration of particles. Given the importance of interstitial flow and extracellular matrix in (viral) particle transport^46^, we additionally developed a microfluidic spheroid-capturing tumor-on-a-chip model that incorporates these elements, as these are absent in the 3D static spheroid cultures.

Analogous microfluidic systems have been developed for the study of drug delivery in 3D tumor cultures, mostly for the evaluation of nanoparticle transport in tumor cultures^47–49^, but without the presence of fibroblasts. We decided to incorporate fibroblasts not only to investigate cell-type specificity, but also to better mimic the physiological cancer environment. Notably, fibroblasts are present in most tumors and have significant effects on the development and properties of *in vivo* tumor spheroids, including the matrix composition and its stiffness and are known to participate in increasing intratumoral interstitial fluid pressure levels, thereby hampering drug delivery^50^.

## Conclusions

This study presents novel insights into the interplay between the transduction by retargeted adenoviruses and the targeted receptor. We demonstrate a receptor-dependent role of the RGD motif at the penton base of adenoviruses and show a lower infectivity of ΔRGD viruses, compared to similar WT adenoviruses, for all receptor-targeted adenoviruses in 3D tumor spheroids. We could show that the decision if ΔRGD adenoviruses are to be preferred for adenoviral vectors for therapeutic applications will ultimately depend on the target receptor, the need for high efficiency versus specificity, as well as the tolerance towards off-target effects. Importantly, the presented insights, generated through combining results from 2D cell culture systems with those from advanced 3D culture systems, will facilitate the rational engineering of novel adenoviral vectors towards specific applications.

## Materials and Methods

### Microfluidic device manufacturing and tumor-on-a-chip culture

Masters of the microfluidic devices were prepared by spin-coating SU8 photoresist (micro resist technology GmbH, Berlin, Germany) on 2-inch silicon wafers (Silicon materials, Pittsburgh, USA). Subsequent processing was done according to the manufacturer's standard protocols^51^. A master for spheroid loading was created in two steps: (1) 20-μm thick film (SU8 2025) was spun, processed and exposed to create cross channels (see the inset of Supplementary Fig. S6); (2) on the top of the undeveloped film another 180-μm thick film (SU8 2100) was spun, processed and exposed; during the second exposure the wafer was aligned to connect the capture chamber and side channels via the cross channels. Finally, the whole construct was developed. A master for the resistance chip was created by processing a single 100-μm thick film (SU8 2100). Schematics of the designs drawn to scale are presented in Supplementary Fig. S6. Devices were subsequently made by soft lithography using polydimethylsiloxane (PDMS; Sylgard-184 silicon elastomer kit, Dow Corning). After curing, PDMS pieces were removed from the master and the inlet and outlet holes were made with a biopsy punch (1.5 mm, Kai Industries). The PDMS pieces were bonded onto a cover glass no. 1.5 by oxygen plasma treatment (Femto plasma system, Diener Electronic). Prior to spheroid loading, the tumor compartment of the microfluidic device was coated with a freshly prepared polydopamine solution (2 mg/ml dopamine hydrochloride (Sigma-Aldrich) in 10 mM Tris-HCl, pH 8.5) for 1 h at room temperature. Spheroids were loaded in a 4 mg/ml collagen solution (Rat tail collagen type I, Corning), prepared via the manufacturer's protocol, using a syringe pump (Harvard apparatus PHD Ultra). Following gelation of the collagen in the tumor compartment for 30 min at room temperature, the device was connected to the syringe with perfusion medium at the inlet of the capillary channel, and to the resistance chip with a tunable resistance (Supplementary Fig. S6) at the outlet of the capillary channel. A representative image of the loaded chip was taken with a ZOE Fluorescent Cell Imager (Bio-Rad). The device was perfused in a 37 ° C incubator for 48 h at a flow rate of 200 μl/h unless indicated otherwise. After the perfusion, the collagen-embedded spheroid culture was washed with PBS, fixed in 4% paraformaldehyde (PFA) in PBS for 30 min and optically cleared using a modified SeeDB protocol^26^, all at room temperature. The SeeDB solution is a refractive-index matching solution and mainly a saturated solution of fructose (80.2% wt/wt) in water with 0.5% α-thioglycerol to prevent the Maillard reaction. For on-chip optical clearing, the tumor compartment was perfused with 29% (w/v) D-(−)-fructose (Sigma-Aldrich) with 0.5% (v/v) α-thioglycerol for 2 h, then with 57.5% D-(−)-fructose/0.5% α-thioglycerol for 5 h, followed by perfusion with 115% D-(−)-fructose/0.5% α-thioglycerol overnight. Perfusion was performed at a flow rate of 400 μl/h. The optically cleared collagen-embedded spheroids were analysed by confocal microscopy.

### Transduction with adenoviral vectors

The DARPin-SHP adapters were expressed as described previously^7^. Adapters contained an N-terminal His_10_-Tag, which was cleaved off by 3 C protease and removed during the purification and was not present in the experiments. Replication-deficient human adenoviruses of serotype 5 (HAdV5) encoding either firefly luciferase or iRFP670 under a CMV promoter were utilized. Both viruses had either a wild-type capsid (WT) with the RGD motif present at the penton base, or a capsid without the RGD motif (ΔRGD). The HAdV5 constructs are derived from the Agilent AdEasy system, and produced and purified as described previously^7^. Labelling of the WT adenovirus with Alexa488 N-hydroxysuccinimide for viral trafficking experiments was performed as described before^7^. Retargeted viral particles were formed by incubating viruses with a calculated 2:1 excess of bispecific DARPin-SHP trimeric adapters (containing the receptor specificity indicated) over adenovirus fiber knobs in HEPES-buffered saline (HBS, 150 mM NaCl, 20 mM HEPES, pH 8.0) for 2 h at room temperature, followed by a 10 min incubation with a 100-fold excess of knob-blocking DARPin 1D3_SHP trimers (not containing any receptor-retargeting DARPin)^14^ (Supplementary Fig. S10), to block any residual free knob which could mediate CAR-based transduction. The retargeted virus was added to cell culture medium to a final concentration of 1,000 viral particles/cell; for viral particle trafficking experiments, the viral particle concentration was 1.0 × 10^9^ viral particles/ml. For transduction of spheroids, the viral particle-containing medium was added in hanging drops. For on-chip transduction, the viral particle-containing medium was perfused with a syringe pump at 1.0 × 10^8^ viral particles/ml.

### Cell culture (monolayer, 3D spheroid), luciferase assay, confocal microscopy, flow cytometry and statistical analyses

For details on the abovementioned procedures, please see the Supplementary Methods section.

### List of Abbreviations

CAR, coxsackievirus and adenovirus receptor; CFSE, carboxyfluorescein succinimidyl ester; DARPin, designed ankyrin repeat protein; ECM, extracellular matrix; EGFR, epidermal growth factor receptor; EpCAM, epithelial cell adhesion molecule; HAdV5, human adenovirus serotype 5; HER2, human epidermal growth factor receptor 2; HER3, human epidermal growth factor receptor 3; PDMS, polydimethylsiloxane.

## Supplementary information

Supplementary information

## Data Availability

All data generated during and/or analysed during the current study are available from the corresponding author on reasonable request.
