# Supplementary material for: Unravelling Receptor and RGD Motif Dependence of Retargeted Adenoviral Vectors using Advanced Tumor Model Systems
Source: Sci Rep. 2019 Dec 6;9:18568. doi: 10.1038/s41598-019-54939-9 (PMC6897923; doi:10.1038/s41598-019-54939-9)
Supplement: Supplementary file 1 — Supplementary information [file 41598_2019_54939_MOESM1_ESM.pdf]

SUPPLEMENTARY INFORMATION

Unravelling Receptor and RGD Motif Dependence of Retargeted Adenoviral Vectors using Advanced Tumor Model Systems

M. Chernyavska, M. Schmid, P.C. Freitag, V. Palacio-Castañeda, A. Piruska, W.T.S. Huck, A. Plückthun and W.P.R. Verdurmen*

***Corresponding author**

SUPPLEMENTARY METHODS

Cell culture

Human skin fibroblasts C5120 were a kind gift of Dr. Rodenburg (Radboud University Medical Center, the Netherlands) and have been extensively characterized before [1]. All other cell lines were obtained from the American Type Culture Collection (ATCC). Human skin carcinoma A-431 cells and human mammary gland adenocarcinoma MCF-7 cells were cultured in Dulbecco's Modified Eagle Medium (DMEM, Gibco, ThermoFisher) supplemented with 10% fetal calf serum (FCS, PAN-Biotech). Human ovarian adenocarcinoma SKOV3 cells were cultured in Roswell Park Memorial Institute (RPMI) 1640 medium (Gibco, ThermoFisher) supplemented with 10% FCS. Human ovarian adenocarcinoma OV-90 cells were cultured in a 1:1 mixture of MCDB 105 (Sigma-Aldrich): Medium 199 (Gibco, ThermoFisher), supplemented with 15% FCS. Human lung adenocarcinoma Calu-3 cells were cultured in Minimal Essential Medium (MEM, Gibco, ThermoFisher) supplemented with 10% FCS. Human skin fibroblasts C5120 and human lung fibroblasts IMR-90 were cultured in Medium 199 supplemented with 10% FCS (Life Technologies) and penicillin-streptomycin (Sigma-Aldrich).

Co-culture spheroid generation and culture

Cells were labelled with the cytoplasmic dyes CellTrace Yellow, CellTrace Far Red and carboxyfluorescein succinimidyl ester (CFSE) (ThermoFisher) according to the manufacturer's instructions. For spheroid preparations, cells were seeded as a co-culture (1,000 cells of each type) in a 30 µl drop of Medium 199 containing 1.2 mg/ml methylcellulose (Sigma-Aldrich) on the inner side of

a Petri dish lid, according to a hanging-drop protocol for spheroid preparation [2]. Following an overnight incubation, spheroids were used in the experiments.

Luciferase assay

The luciferase assay was performed with the firefly luciferase reporter assay system (Promega Corp.) according to the manufacturer's instructions. A total of 1.0 × 10^4 cells were seeded per well in a 96-well cell culture plate (Corning) and incubated overnight. Following the overnight culture, cells were transduced as described and incubated for 48 h. Luciferase activity was measured using a Synergy™ 2 Multi-Mode Microplate Reader (BioTek Instruments, Inc.). All assays were carried out in biological duplicates. The number of independent experiments is indicated in the figure caption. The data was normalized over the sum of all signal intensities per experiment.

Flow cytometry

The investigation of viral transduction efficiency and specificity by flow cytometry was done via the detection of iRFP670 in cells pre-labelled with cytoplasmic dyes using the following lasers: 488 nm for carboxyfluorescein (detection: 525/50 nm) and CellTrace Yellow (detection: 585/40 nm), and 635 nm for iRFP670 (detection: 655-730 nm). Cells cultured in 2D were trypsinized while spheroids were dissociated by extensive washing in PBS and trypsinization for 15 min at 37°C. Cells were fixed in 4% PFA in PBS for 10 min and resuspended in PBS. Flow cytometry was performed using a MacsQuant Analyzer 10 flow cytometer (Miltenyi Biotec). Tumor cells and fibroblasts in co-cultures were discriminated via their cytoplasmic dyes. The relative transduction efficiency was determined after normalizing over the sum of percentages of positive cells per experiment in order to control for overall virus activity.

For quantification of the absolute number of the cell surface receptors EpCAM and EGFR, BD QuantiBRITE PE (Phycoerythrin) beads (PE Fluorescence Quantitation Kit, BD Biosciences) were used, which have known numbers of surface PE molecules per bead (information provided by the manufacturer). Briefly, tumor cells and fibroblasts cultured in 2D were detached with 10 mM EDTA and

stained with PE-conjugated mouse anti-EGFR antibody (BioLegend, cat. no. 352903) and PE-conjugated mouse anti-EpCAM antibody (Abcam, cat. no. ab112068). PE-conjugated beads and stained cells were analyzed by flow cytometry using using a MacsQuant Analyzer 10 flow cytometer (Miltenyi Biotec), by excitation of PE with 488 nm and detection of PE fluorescence (detection: 585/40 nm). Using the provided values of number of PE molecules per bead, a calibration curve was generated using the measured median fluorescence intensity values of the PE beads. The flow cytometry data was analyzed with FlowJo 10.0.8rl.

Confocal microscopy

For confocal microscopy, cells in 2D were seeded, incubated and analyzed in µ-slide 8-well chambers (Ibidi, Gräfelfing, Germany). For imaging of 3D spheroid co-cultures, spheroids were collected, washed in PBS and fixed as described above, and embedded in a 15-µl drop of 4 mg/ml collagen in an 8-well Ibidi chamber. Collagen gelation was achieved by incubation at room temperature for 10 min followed by incubation at 37°C for 30 min. The embedded spheroids were optically cleared as described above, always using 250 µl solution per well. For the co-cultures in the microfluidic devices, images of spheroids were acquired on-chip. For image acquisition, a Leica TCS SP8 confocal microscope (Leica Microsystem SP8) was used. Carboxyfluorescein was excited at 488 nm (detection: 496-550 nm), CellTrace Yellow at 549 nm (detection: 586-679 nm) and iRFP670 was excited at 670 nm (detection: 679-795 nm). CellTrace Far Red was excited with 633 nm (detection: 641-711 nm). The following objectives were used: for 2D: HC PL FLUOTAR 20x/0.50 dry and HCX APO U-V-I 40x/0.75 DRY; for 3D image acquisition: HCX PL APO 10x/0.40 dry and HC PL FLUOTAR 20x/0.50 dry. The collected images were processed and quantified using Fiji image analysis software [3].

The method used for quantification of transduced tumor cells and fibroblasts from confocal microscopy images was validated against results from flow cytometry. Co-cultures were transduced and analyzed via flow cytometry as described above. Corresponding confocal images were analyzed with Fiji software: all channels from the microscopy were subjected to a Gaussian blur, followed by

thresholding to create masks of 'tumor cells', 'fibroblasts' and 'transduced cells'. The fraction of overlapping areas of either 'fibroblasts' or 'tumor cells' with 'transduced cells' was determined and taken as an approximate measure of the percentage of transduced cells.

Statistical analysis

All statistical analyses were carried out using GraphPad Prism version 5.03. P values < 0.05 were considered significant. For comparisons between two groups, two-tailed unpaired t-tests were performed, assuming normally distributed data. For comparisons between multiple groups and two independent factors (e.g. virus and cell line for the luciferase assay; culture condition [2D or 3D] and virus-retargeting approach for flow cytometry), two-way ANOVAs with Bonferroni post hoc tests were utilized.

SUPPLEMENTARY FIGURES

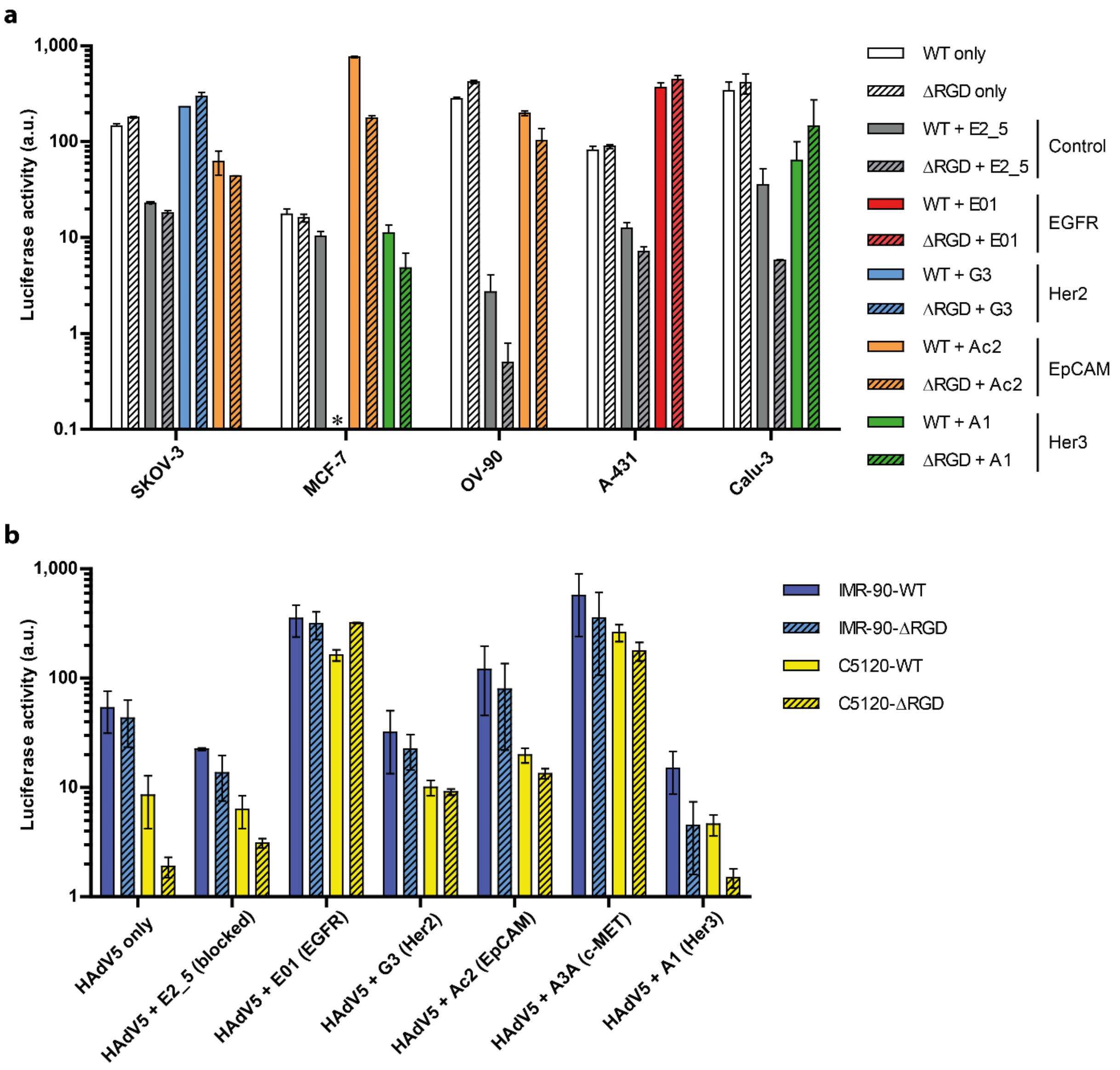

Supplementary Figure S1. Transduction efficiency of retargeted HAdV5 in tumor cell lines and primary fibroblasts in 2D monocultures. (a) Tumor cell lines were evaluated for their capacity to be transduced with HAdV5 with (WT) or without the RGD motif (ΔRGD), retargeted to a specific receptor. HAdV5 only (i.e. CAR-directed) and HAdV5 complexed with the non-binding DARPin E2_5 (i.e. knob-blocked) were used as controls. (b) IMR-90 and C5120 fibroblasts were evaluated as in (a) for their capacity to be transduced with HAdV5 with (WT) or without the RGD motif (ΔRGD). Two independent experiments were performed. Each experiment contained two biological replicates. Mean values are shown, including range for the mean values per independent experiment. *below detection threshold. HAdV5, human adenovirus serotype 5.

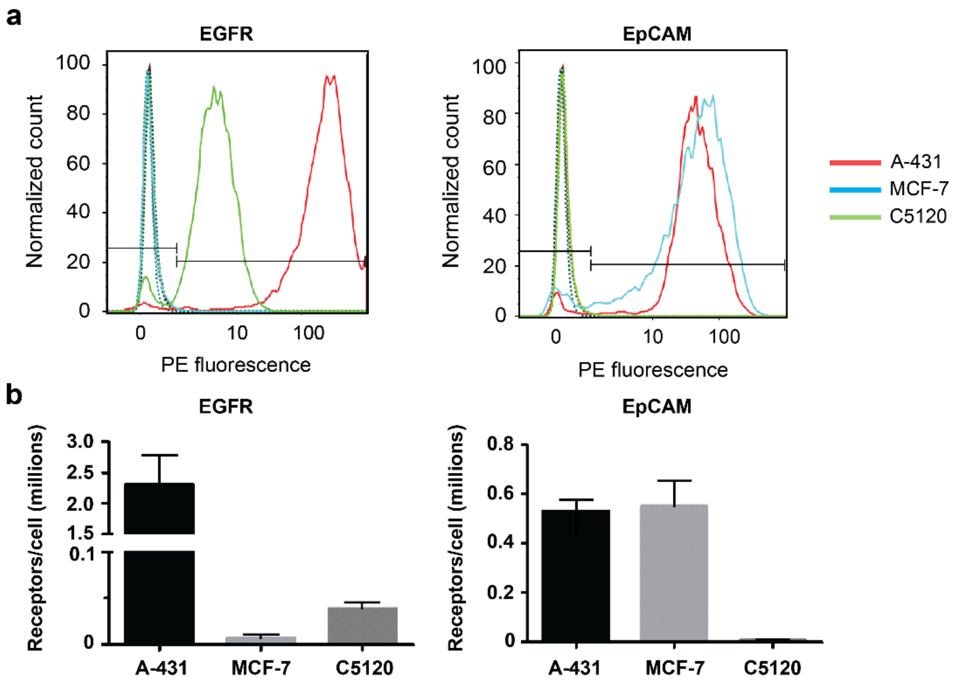

Supplementary Figure S2. Quantification of EGFR and EpCAM on the surface of A-431, MCF-7 and C5120 cell lines. (a) Overlay histogram plots of the normalized cell count of cells stained with PE (phycoerythrin)-labelled anti-EGFR or anti-EpCAM antibodies. Antibody signals are shown with continuous lines. Dotted lines of the same color represent the corresponding isotype control. Representative histogram plots are shown, n = 3. (b) Flow cytometry-based quantification of the absolute number of EGFR and EpCAM molecules on the cell surface. A calibration curve obtained with beads coated with known amounts of PE molecules was used to derive absolute numbers. Bars indicate the absolute number of receptors per cell ± s.e.m., n = 3. EGFR, epidermal growth factor receptor; EpCAM, epithelial cell adhesion molecule.

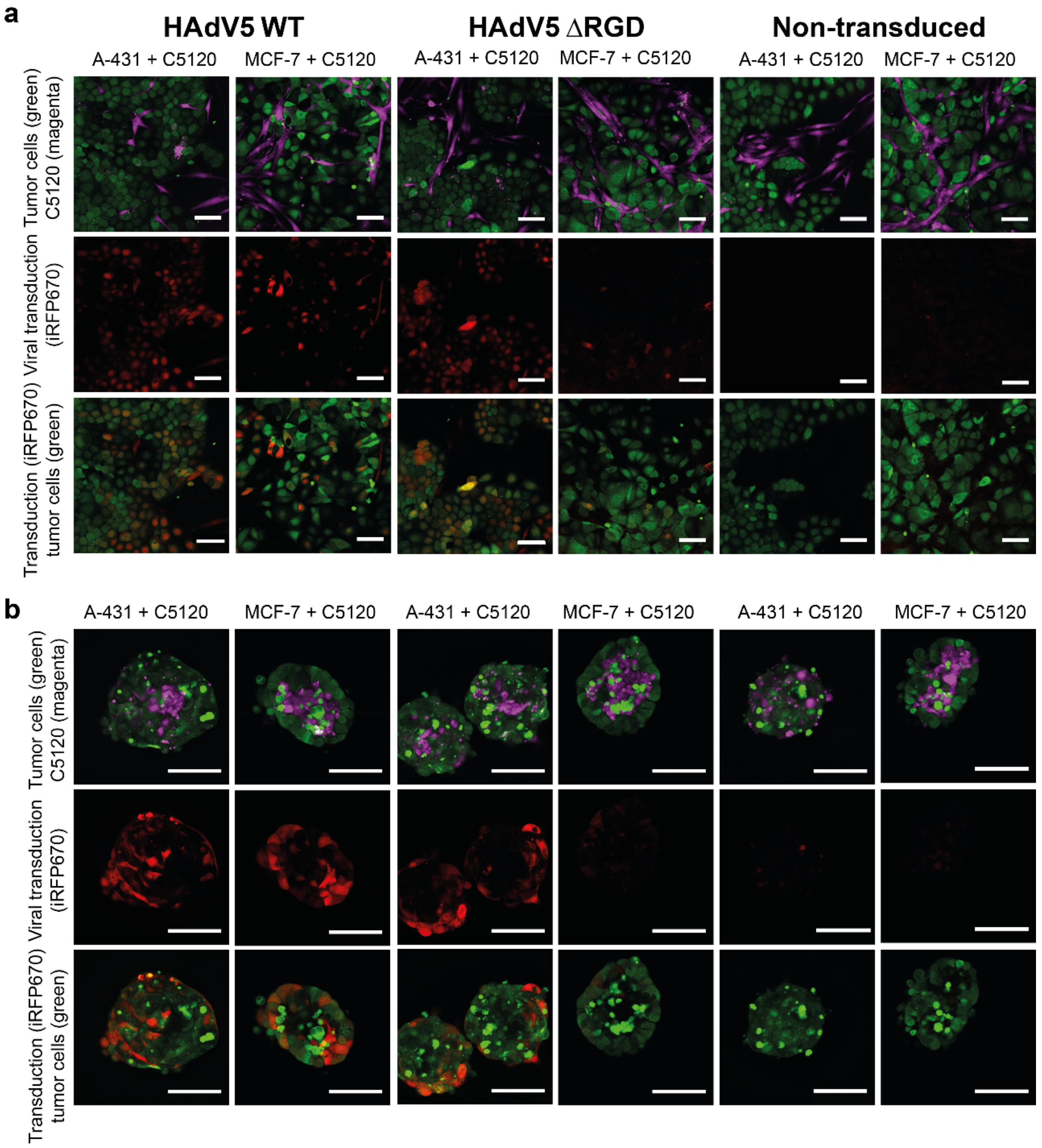

Supplementary Figure S3. Confocal microscopy of tumor cells and fibroblasts transduced with WT and ΔRGD HAdV5. Confocal slices from the centre of EGFR-retargeted (A-431, via E01 adapter) and EpCAM-retargeted (MCF-7, via Ac2 adapter) HAdV5 transduction in 2D (a) and 3D (b) are shown. C5120 fibroblasts were labelled with CellTrace Yellow (magenta), A-431 and MCF-7 cells were labelled with CFSE (green). iRFP670 (red) was used for detection of the virus-transduced cells. Representative images are shown from two independent experiments, with three spheroids analyzed per condition per experiment. The scale bars are 100 μm.

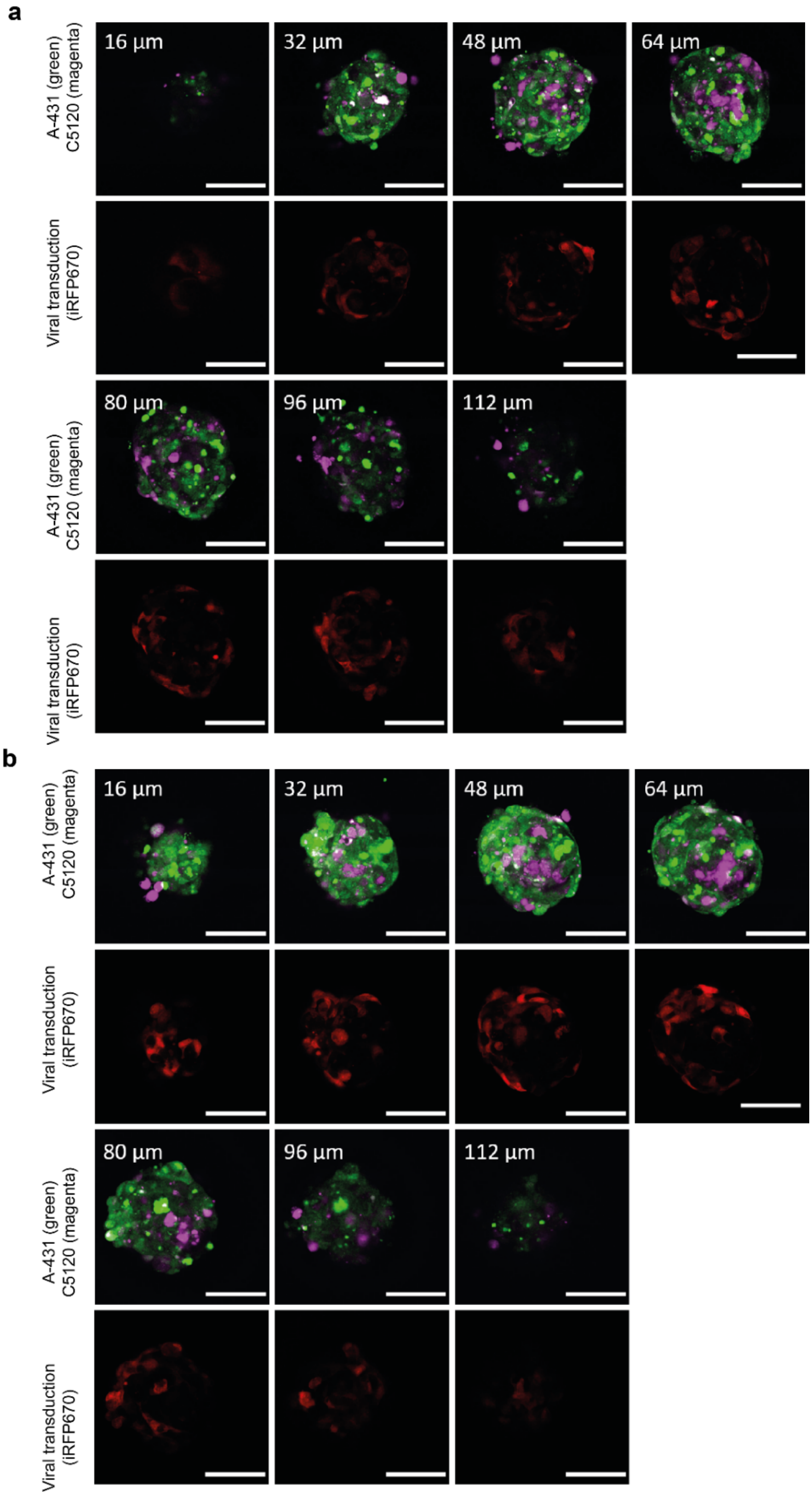

Supplementary Figure S4. Z-stacks of optically cleared tumor spheroids. Individual slices of z-stacks obtained with confocal microscopy of optically cleared spheroids transduced with HAdV5. C5120 fibroblasts were labelled with CellTrace Yellow (magenta). Tumor cells were labelled with CFSE (green). iRFP670 (red) was used for detection of virus-transduced cells. Relative z-positions from the bottom of the spheroid are indicated. Representative Z-stacks are shown from two independent experiments. The scale bars are 100 µm**. Panel S4.1.** (a) A-431_C5120 spheroid transduced with naked HAdV5. (b) A-431_C5120 spheroid transduced with knob-blocked HAdV5 (retargeted with DARPin E2_5).

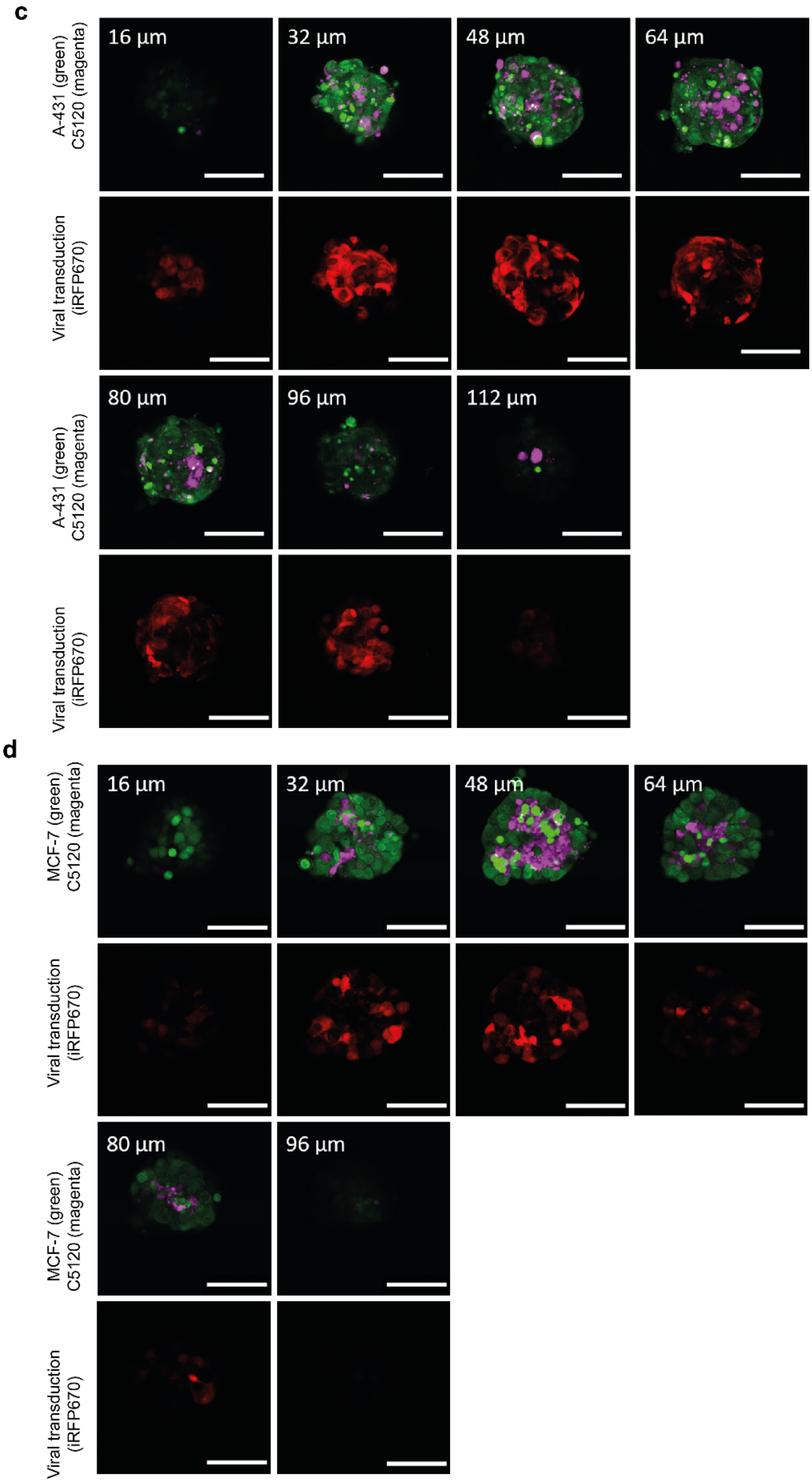

Supplementary Figure S4 (continued), Panel S4.2. (c) A-431_C5120 spheroid transduced with EGFR-retargeted HAdV5. (d) MCF-7_C5120 spheroid transduced with naked HAdV5.

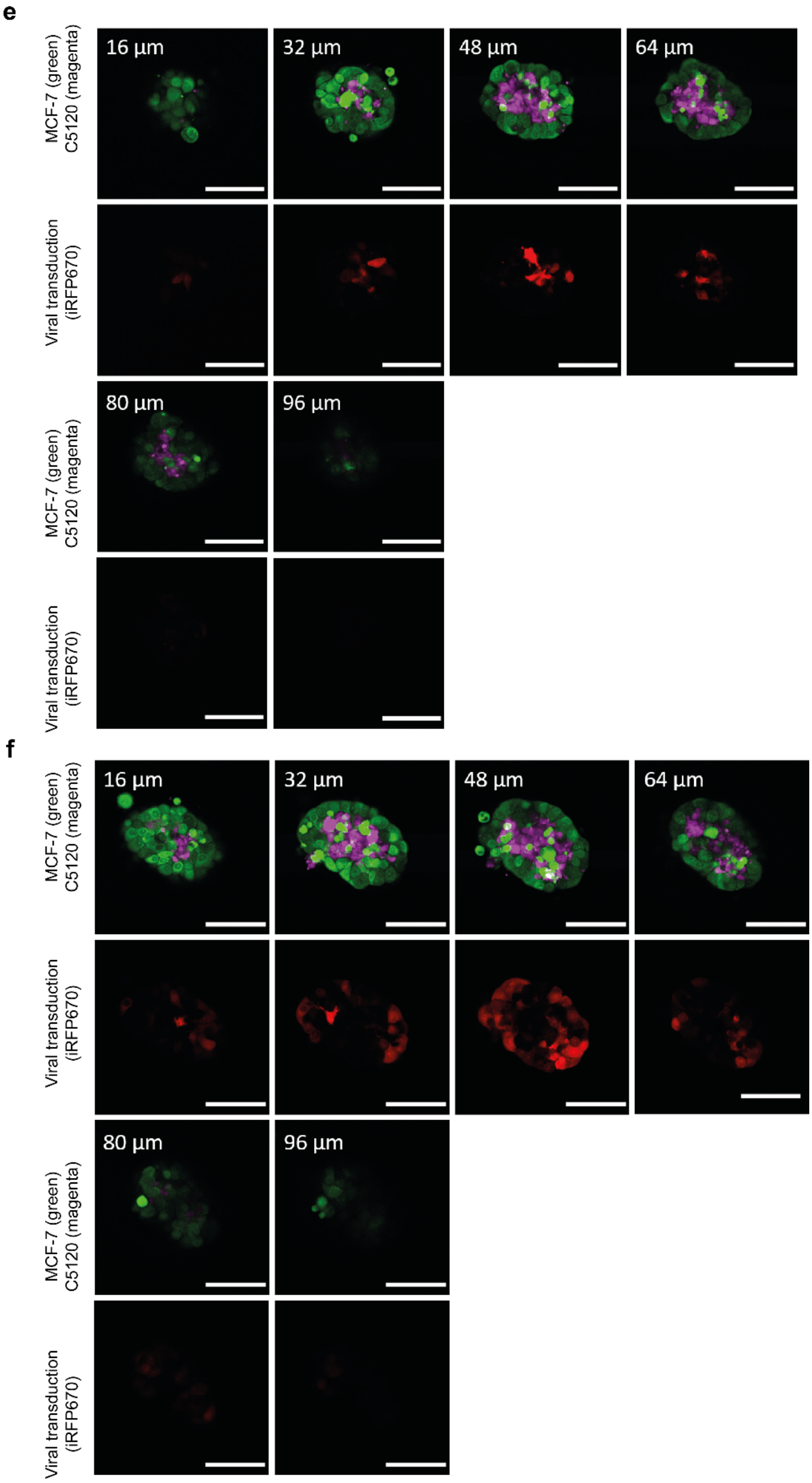

Supplementary Figure S4 (continued), Panel S4.3. (e) MCF-7_C5120 spheroid transduced with knob-blocked HAdV5 (retargeted with DARPin E2_5). (f) MCF-7_C5120 spheroid transduced with EpCAM-retargeted HAdV5.

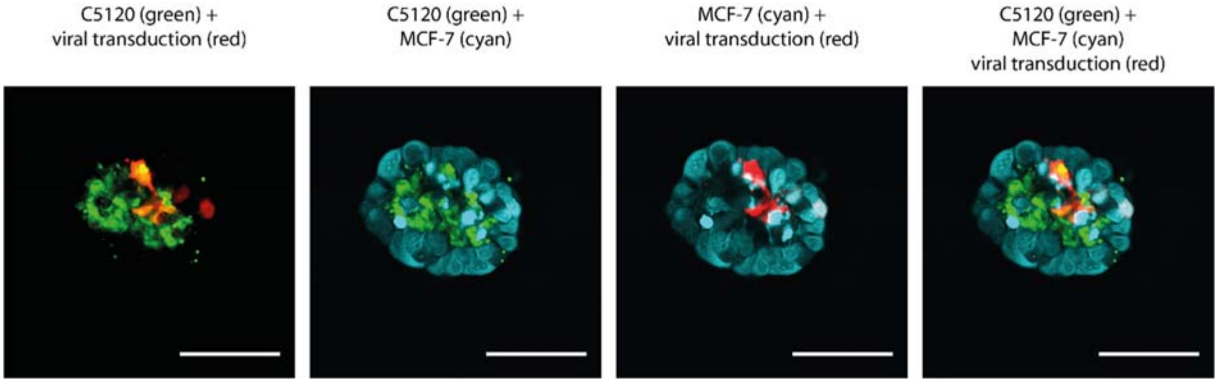

Supplementary Figure S5. Transduction of a co-culture spheroid consisting of MCF-7 and C5120 cells. The control adapter E2_5 (which blocks receptor binding) was used as an adapter on WT HAdV5. It can be observed that transduction (red, iRFP670) is preferentially localized in the core of the spheroid in C5120 fibroblasts (green), whereas MCF-7 cells (cyan) remain mostly not transduced. Viral transduction is depicted in red. A slice in the middle of an optically cleared spheroid is shown. The scale bar corresponds to 100 μm. A representative image is shown from two independent experiments with three spheroids analyzed per condition per experiment.

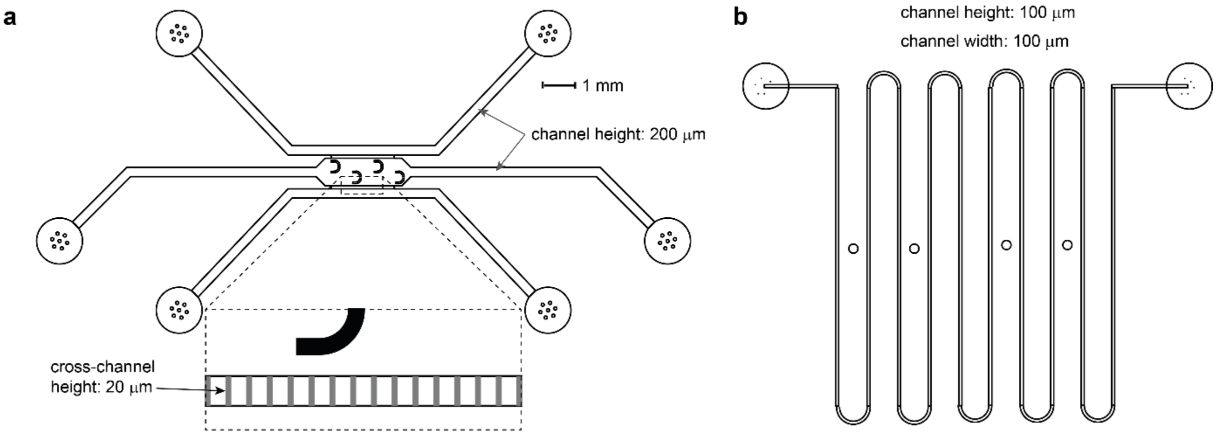

Supplementary Figure S6. Designs of microfluidic chip drawn to scale. (a) Layout of the microfluidic chip for capturing spheroids. Circles indicate in- and outlets for perfusion and/or loading collagen-embedded spheroids. Four 300 μm-sized bowl-shaped elements were included for capturing spheroids in the device. A lower layer of 20 μm (height) containing cross channels (length: 100 μm, width: 20 μm) separated by 50 μm-sized pillars was fabricated to connect the main middle compartment, containing the spheroid-capturing elements, with the side channels. A subsequent layer of 180 μm (height) was fabricated, yielding a total channel height of 200 μm. (b) Layout of the resistance channel chip that was connected via tubing to the spheroid-capturing chip in order to control the hydrodynamic resistance and thereby the interstitial flow. Through punching a hole at the desired location in the resistance channel chip (i.e. by not utilizing the full length of the channel system), the hydrodynamic resistance can be controlled, thereby affecting the interstitial flow rate.

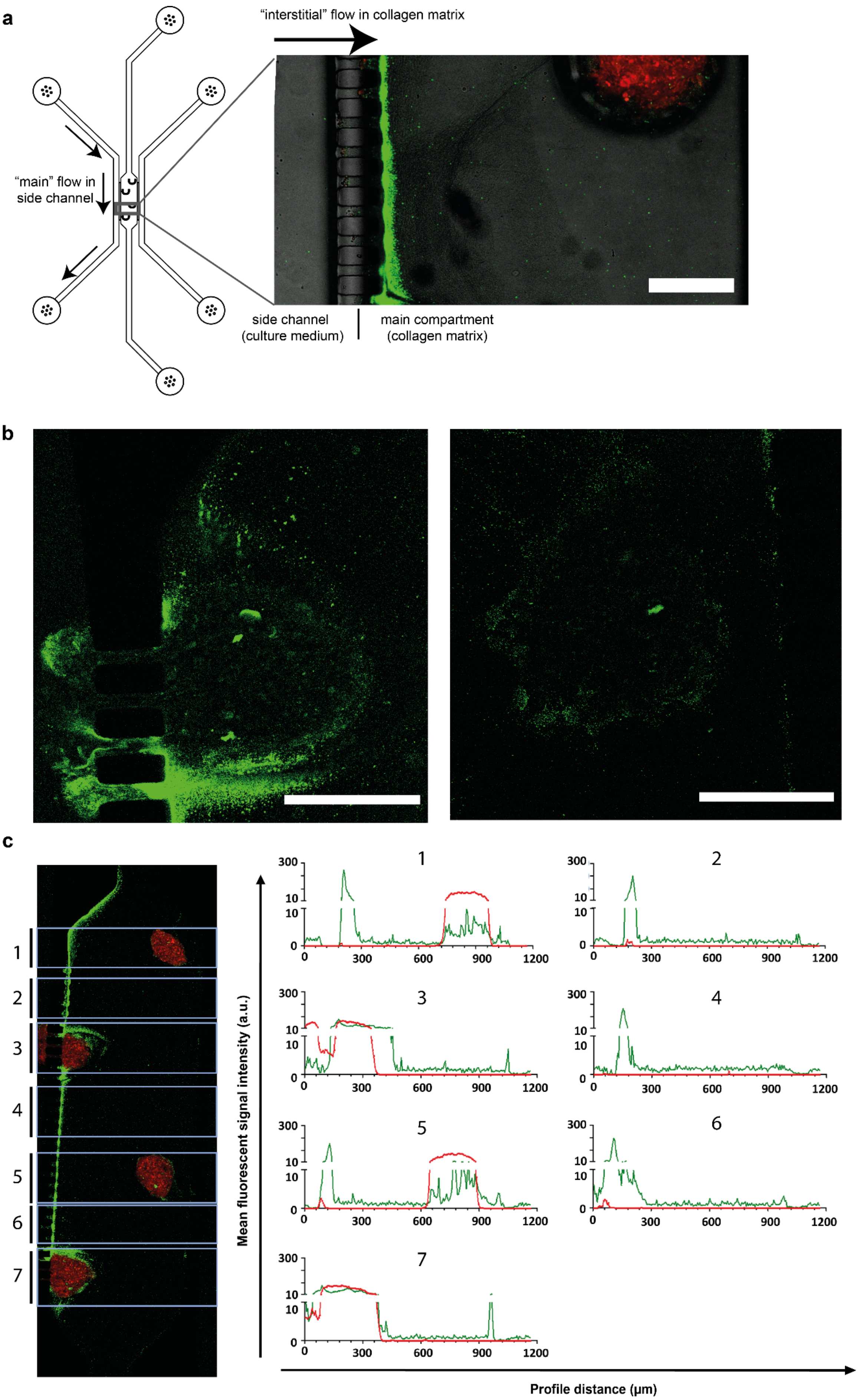
a
"interstitial" flow in collagen matrix
"main" flow in
side channel
side channel
(culture medium)
main compartment
(collagen matrix)
b
c
1
2
3
4
5
6
7
Mean fluorescent signal intensity (a.u.)
Profile distance (µm)
300
10
10
0
0
300
600
900
1200

Supplementary Figure S7. Distribution of Alexa Fluor 488-labelled, EGFR-retargeted adenoviral particles. (a) A confocal microscopy image of the microfluidic tumor-on-a-chip perfused with Alexa Fluor 488-labelled viral particles is shown. Viral particles (green) accumulate at the interface between the medium and the collagen in the side channel, forming a thick ridge of viral particles. Beyond this ridge, a more even distribution of the viral particles is observed, indicating a good mobility of the viral particles in the collagen matrix once the initial interface has been crossed. Tumor cells were stained with CellTrace Far Red. An overlay image of transmitted image and green (viral particles) and red (tumors cells) is depicted. A representative image from two independent experiments is shown. The scale bar is 200 μm. (b) Magnified images of the enrichment of EGFR-retargeted viral particles around the spheroids, as shown in Fig. 4c. The scale bars are 200 μm. (c) Intensity profiles of the fluorescent signal distribution (Alexa Fluor 488 and CellTrace Red) in the collagen compartment were generated in seven regions of interest (ROI) as indicated in the overlay microscopy image. The peaks of Alexa Fluor 488 at 180-300 µm profile distance correspond to the 'filter cake' particle accumulation at the culture medium-collagen interface. In ROI 1,3,5 and 7 the overlapping peaks of Alexa Fluor 488 with CellTrace Red correspond to spheroid regions where viral particles are enriched.

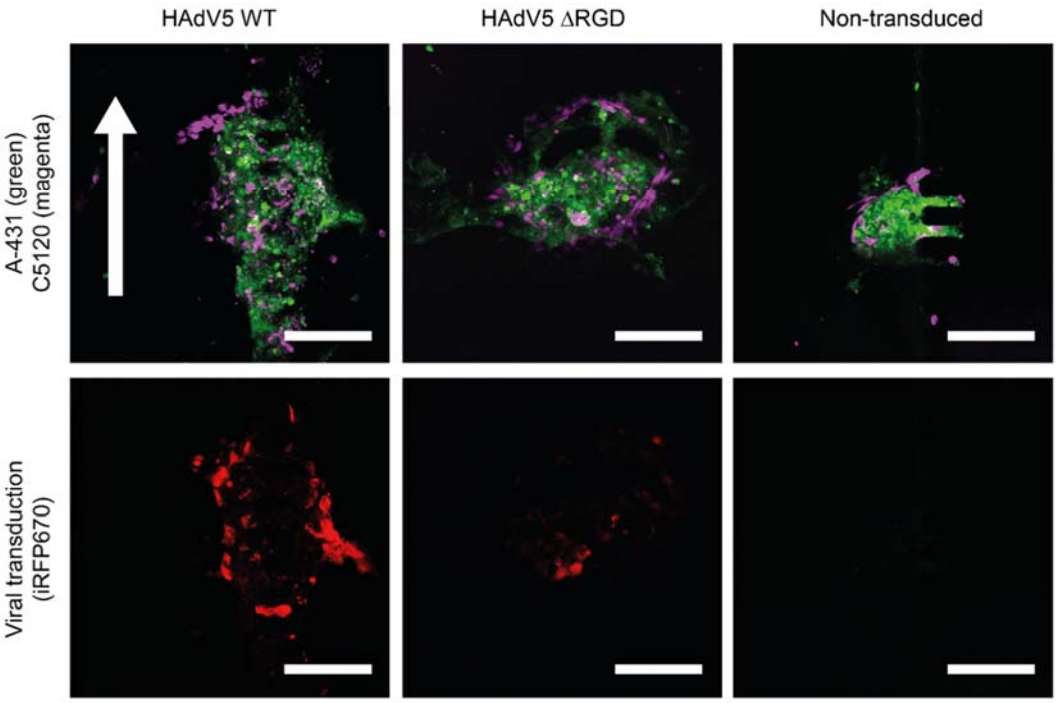

Supplementary Figure S8. On-chip transduction of tumor spheroids with retargeted HAdV5 with or without the RGD motif. Confocal microscopy images of the on-chip optically cleared tumor spheroids transduced with the EGFR-retargeted adenovirus or a non-transduced control are shown. C5120 fibroblasts were labelled with CellTrace Yellow (magenta), A-431 cells were labelled with CFSE (green). iRFP670 (red) was used for detection of viral transduction. Representative images are shown, n = 3. The white arrow in the upper left image indicates the direction of flow. The scale bar indicates 200 µm. EGFR, epidermal growth factor receptor.

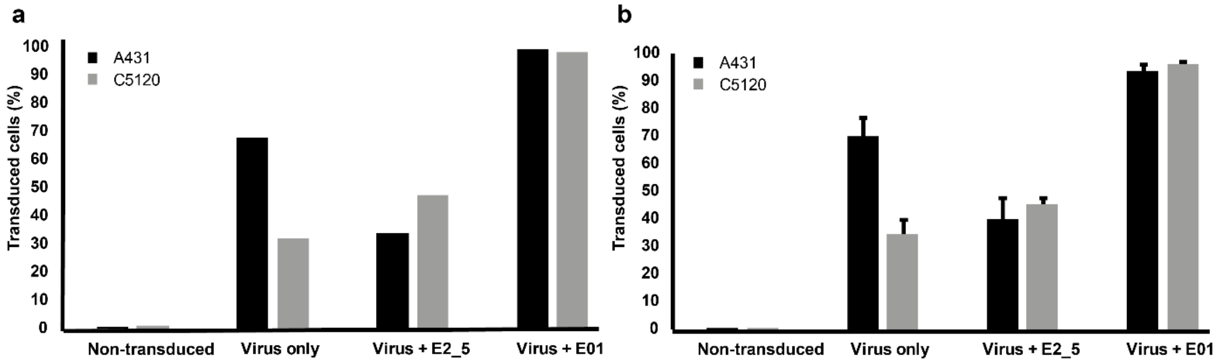

Supplementary Figure S9. Validation of image-based quantification of confocal microscopy data by comparison with flow cytometry. Co-cultures of A-431 and C5120 were transduced with WT adenoviruses without adapter (virus only), with E2_5 (does not bind other proteins) or E01 (binds EGFR). Cells were either trypsinized and analyzed by flow cytometry (a) or imaged via confocal microscopy (b). Confocal microscopy images were analyzed by Fiji software as described in Materials and Methods. 'Virus' refers to HAdV5. For the analysis of microscopy images, error bars reflect range between two independent experiments, where at least two technical replicates were analyzed per condition per experiment. EGFR, epidermal growth factor receptor.

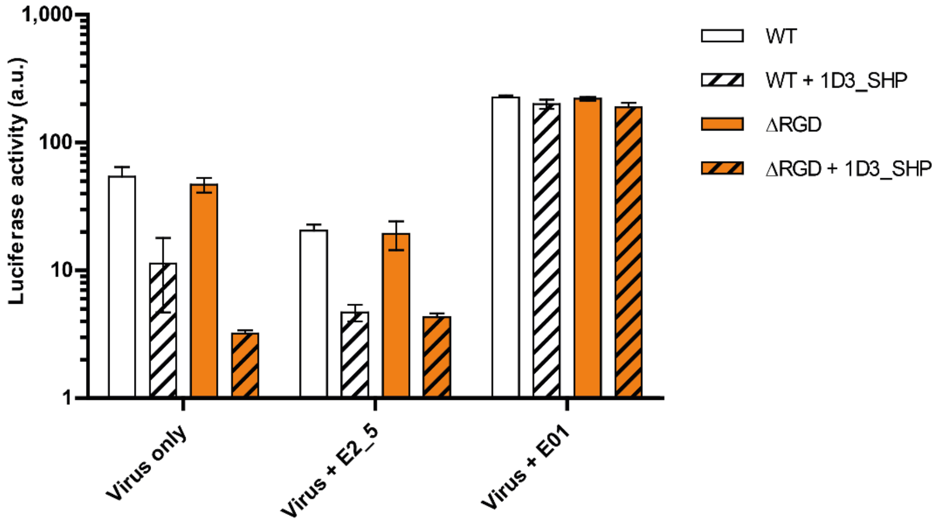

Supplementary Figure S10. Effect of addition of excess knob-blocking DARPin-SHP trimers after formation of retargeted adenoviral complexes. After adding the 1D3_SHP-fused retargeting DARPin E01 (binds EGFR) or the control DARPin E2_5 (does not bind any external protein) to the adenoviral vector, addition of 100-fold excess of 'naked' 1D3_SHP (no retargeting DARPin) further reduces transduction especially of the negative control E2_5, indicating the coating was not 100% complete (leaving some uncovered knob able to interact with CAR), and the non-retargeted control labelled 'virus only'. The addition of a 100-fold excess of 1D3_SHP was included as a standard step in the preparation of retargeted viruses. Two independent experiments were performed with each two biological replicates. Error bars reflect the range between the means of the independent experiments. Virus refers to 'HAdV5'; 1D3, knob-binding DARPin; EGFR, epidermal growth factor receptor.

SUPPLEMENTARY TABLES

Supplementary Table S1. References for confirmed receptor overexpression for investigated cell lines

Cell line	Overexpressed receptor [reference]
SKOV-3	EpCAM [4] and Her2 [5]
MCF-7	EpCAM [6] and Her3 [7]
OV90	EpCAM [4]
A-431	EGFR [8]
Calu-3	Her3 [7]

EpCAM, epithelial cell adhesion molecule; EGFR, epidermal growth factor receptor; Her2, human epidermal growth factor receptor 2; Her3, human epidermal growth factor receptor 3

Supplementary Table S2. Viral transduction of C5120 fibroblasts by ∆RGD viruses encoding iRFP670 in 2D monolayers

	**Relative transduction efficiency (a.u.) ± s.e.m.**		
**∆RGD - 2D**	C5120 (mono-culture)	C5120 (A-431 co-culture)	C5120 (MCF-7 co-culture)
**Non-transduced**	0.82 ± 0.18	1.42 ± 0.32	1.07 ± 0.37
**Virus only**	23.30 ± 1.99	34.99 ± 14.71	10.21 ± 1.97
**Virus + E2_5 (control)**	25.42 ± 5.76	27.19 ± 11.15	15.99 ± 5.65
**Virus + E01 (α-EGFR)**	109.34 ± 23.99	79.45 ± 8.97	83.37 ± 11.74
**Virus + Ac2 (α-EpCAM)**	38.76 ± 11.86	21.65 ± 9.90	37.78 ± 13.67

MOI was 1,000 viral particles/cell, n=3

Supplementary Table S3. Transduction of C5120 fibroblasts via WT and ∆RGD viruses encoding iRFP670 in 3D tumor spheroids

	Relative transduction efficiency (a.u.) ± s.e.m.	
**WT - 3D**	C5120 (A-431 co-culture)	C5120 (MCF-7 co-culture)
**Non-Transduced**	1.30 ± 0.38	0.94 ± 0.30
**Virus only**	4.99 ± 2.58	3.07 ± 1.06
**Virus + E2_5 (control)**	3.98 ± 1.12	3.17 ± 1.34
**Virus + E01 (α-EGFR)**	7.44 ± 3.37	7.95 ± 4.08
**Virus + Ac2 (α-EpCAM)**	7.79 ± 3.22	5.36 ± 1.66
**∆RGD - 3D**		
**Non-Transduced**	1.29 ± 0.31	0.91 ± 0.23
**Virus only**	3.59 ± 1.10	1.04 ± 0.11
**Virus + E2_5 (control)**	3.48 ± 1.06	1.21 ± 0.46
**Virus + E01 (α-EGFR)**	3.14 ± 0.59	8.05 ± 4.58
**Virus + Ac2 (α-EpCAM)**	2.55 ± 0.31	1.73 ± 0.20

MOI was 1,000 viral particles/cell, n=3

REFERENCES

1. Distelmaier, F. *et al.* Trolox-sensitive reactive oxygen species regulate mitochondrial morphology, oxidative phosphorylation and cytosolic calcium handling in healthy cells. *Antioxid Redox Signal* **17**, 1657-1669 (2012).
2. Foty, R. A simple hanging drop cell culture protocol for generation of 3D spheroids. *J Vis Exp*, 2720 (2011).
3. Schindelin, J. *et al.* Fiji: an open-source platform for biological-image analysis. *Nat Methods* **9**, 676-682 (2012).
4. Beaufort, C. M. *et al.* Ovarian cancer cell line panel (OCCP): clinical importance of in vitro morphological subtypes. *PLoS One* **9**, e103988 (2014).
5. Magnifico, A. *et al.* Tumor-initiating cells of HER2-positive carcinoma cell lines express the highest oncoprotein levels and are sensitive to trastuzumab. *Clin. Cancer Res.* **15**, 2010-2021 (2009).
6. Osta, W. A. *et al.* EpCAM is overexpressed in breast cancer and is a potential target for breast cancer gene therapy. *Cancer Res.* **64**, 5818-5824 (2004).
7. Takagi, S. *et al.* HER2 and HER3 cooperatively regulate cancer cell growth and determine sensitivity to the novel investigational EGFR/HER2 kinase inhibitor TAK-285. *Oncoscience* **1**, 196-204 (2014).
8. Zhang, F. *et al.* Quantification of epidermal growth factor receptor expression level and binding kinetics on cell surfaces by surface plasmon resonance imaging. *Anal. Chem.* **87**, 9960-9965 (2015).
